# Phosphorylated and Phosphonated Low‐Complexity Protein Segments for Biomimetic Mineralization and Repair of Tooth Enamel

**DOI:** 10.1002/advs.202103829

**Published:** 2022-01-02

**Authors:** Rong Chang, Yang‐Jia Liu, Yun‐Lai Zhang, Shi‐Ying Zhang, Bei‐Bei Han, Feng Chen, Yong‐Xiang Chen

**Affiliations:** ^1^ Key Laboratory of Bioorganic Phosphorus Chemistry and Chemical Biology (Ministry of Education) Department of Chemistry Tsinghua University Beijing 100084 China; ^2^ Central Laboratory Peking University Hospital of Stomatology Beijing 100081 China

**Keywords:** low‐complexity protein segments, mineralization, phosphonated, phosphorylated, tooth enamel

## Abstract

Biomimetic mineralization based on self‐assembly has made great progress, providing bottom‐up strategies for the construction of new organic–inorganic hybrid materials applied in the treatment of hard tissue defects. Herein, inspired by the cooperative effects of key components in biomineralization microenvironments, a new type of biocompatible peptide scaffold based on flexibly self‐assembling low‐complexity protein segments (LCPSs) containing phosphate or phosphonate groups is developed. These LCPSs can retard the transformation of amorphous calcium phosphate into hydroxyapatite (HAP), leading to merged mineralization structures. Moreover, the application of phosphonated LCPS over phosphorylated LCPS can prevent hydrolysis by phosphatases that are enriched in extracellular mineralization microenvironments. After being coated on the etched tooth enamel, these LCPSs facilitate the growth of HAP to generate new enamel layers comparable to the natural layers and mitigate the adhesion of *Streptococcus mutans*. In addition, they can effectively stimulate the differentiation pathways of osteoblasts. These results shed light on the potential biomedical applications of two LCPSs in hard tissue repair.

## Graphical abstract

Herein, inspired by the cooperative effects of key components in biomineralization microenvironments, a new type of biocompatible peptide scaffold based on flexibly self‐assembling low‐complexity protein segments (LCPSs) containing phosphate or phosphonate group is developed. These LCPSs can mediate to form merged mineralization structures leading to enamel remineralizaiton, potentially antibacterial adhesion, and stimulation of osteogenic differentiation.

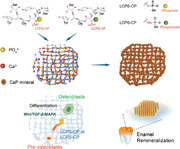

## 1 Introduction

Biomimetic mineralization based on self‐assembly is important for the formation of highly mineralized organic–inorganic hybrid complexes, which have great potential for use in elucidating the mechanism of mineralization,^[^ ^1^ ^]^ constructing new biomineral‐based materials,^[^ ^2^ ^]^ and treating human bone defects and dental decay.^[^ ^3^ ^]^ Biomineralization of bone and teeth plays an important role in biological regeneration and commonly involves complex ingredients, including: i) collagen or amelogenin, which function as the primary matrix to induce the formation of calcium phosphate (CaP);^[^ ^4^ ^]^ ii) non‐collagenous proteins (NCPs), which are often hyperphosphorylated proteins^[^ ^5^ ^]^ and other small molecules that serve as additives^[^ ^6^ ^]^ to regulate biomineralization; and iii) various cells, such as osteoblasts, that fine‐tune extracellular secretion of organic substances and intracellular signaling pathways related to mineralization.^[^ ^7^ ^]^ Accordingly, diverse biomimetic self‐assembly system components, such as polymers,^[^ ^2^, ^8^ ^]^ proteins,^[^ ^3^, ^9^ ^]^ peptides,^[^ ^10^ ^]^ saccharides,^[^ ^11^ ^]^ and DNA^[^ ^12^ ^]^ have been developed for biomimetic mineralization. Nevertheless, due to the complexity of the biomineralization process as well as the clinical need for long‐lasting materials, effective biomimetic mineralized scaffolds combining the cooperative effects of matrixes, additives, and cell stimulation in the biomineralization microenvironment are still needed.

The existing strategies usually implement stably self‐assembling scaffolds with relatively rigid structures that can faithfully mimic the template effect of collagen or amelogenin but often lack the regulatory effects of flexible NCPs and other additives. In addition, the existence of tyrosine‐rich motif in amelogenin^[^ ^1g^ ^]^ inspired us to look for self‐assembly peptide sequences containing both rich tyrosine and phosphate modification, which might be competitive in mediating tooth remineralizaiton. Recently, it was revealed that the low‐complexity protein segment (LCPS) ^37^SYSGYS^42^ of the fused in sarcoma (FUS) protein containing two tyrosine residues can stack to form kinked structures and reversible amyloid fibrils, termed low‐complexity aromatic‐rich kinked segments (LARKS).^[^ ^13^ ^]^ This LCPS possesses significant features distinct from those of other typical self‐assembling peptides, including: i) high aqueous solubility due to the main hydrophilic amino acids, ii) structural flexibility contributed by glycine, and iii) an ability to engage in molecular entanglement and acquire a network morphology due to weak multivalent interactions.^[^ ^13b^ ^]^ In addition, this LCPS maintains its self‐assembly ability even after phosphorylation at Ser^39^.^[^ ^13a^ ^]^ Thus, the phosphorylated LCPS from the FUS protein with moderate self‐assembly ability is a potential biomineralization scaffold that could combine the synergistic effects of a template and a regulatory additive.

However, phosphate anchored on this LCPS can be easily hydrolyzed by the abundant alkaline phosphatase (ALP) enriched in the biomineralization microenvironment, which might greatly attenuate its ability to direct calcification alongside peptide assembly in a controllable manner.^[^ ^14^ ^]^ A phosphonated mimetic (cpS) has been developed as a phosphatase‐inert replacement of phosphorylated Ser (pS) and applied in the synthesis of peptides and proteins.^[^ ^15^ ^]^ Interestingly, we noticed that bisphosphonates, a class of commercial drugs for the treatment of osteoporosis and similar diseases, can promote the proliferation of osteogenesis‐related cells and biosynthesis of collagen by bone cells.^[^ ^16^ ^]^ Thus, incorporation of a phosphonate group into LCPSs might not only create a nonhydrolysable phosphate mimetic for mineralization but also enable stimulation of osteogenic cells.

**Figure 1:**
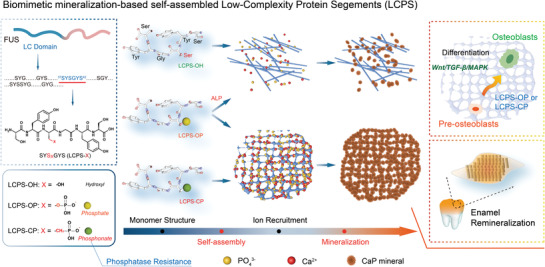
Schematic illustration of phosphorylated and phosphonated LCPSs for biomimetic mineralization and repair of tooth enamel.

In this study, we developed a new type of biomimetic scaffold for biomineralization based on flexibly self‐assembling LCPSs containing a phosphate or phosphonate group (named LCPS‐OP and LCPS‐CP, respectively; the unmodified scaffold is LCPS‐OH) that regulated and promoted mineralization (**Figure** **1**). Moreover, coating LCPS‐OP and LCPS‐CP on the surface of etched tooth enamel facilitates the epitaxial growth of hydroxyapatite (HAP) to generate repaired enamel comparable to natural enamel and mitigates the adhesion of *Streptococcus mutans*. In addition, we found that both LCPS‐OP and LCPS‐CP could stimulate osteogenic differentiation by activating some mineralization‐associated genes involved in many essential signaling pathways.

## 2 Results

### 2.1 Synthesis of LCPSs and Characterization of Their Assembly

**Figure 2:**
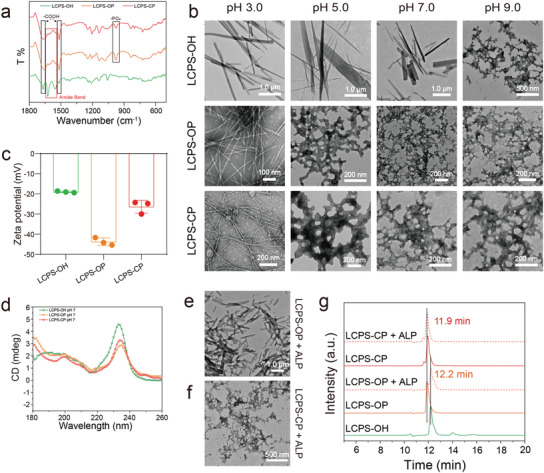
Characterization of LCPSs and their self‐assembly. a) FTIR characterization of different molecular structures among the LCPS peptides with a concentration of 10 mg mL^−1^ LCPS assembly solution. b) Negatively stained TEM images showing the self‐assembled structures of LCPS‐OH, LCPS‐OP, and LCPS‐CP at pH 3.0, 5.0, 7.0, and 9.0. c) Zeta potential values of LCPSs at pH 7. The data are the means ± SD (*n* = 3 repeats per group). d) CD spectra of LCPS‐OH, LCPS‐OP, and LCPS‐CP assembly solutions (10 mg mL^−1^) at pH 7. Self‐assembly of e) LCPS‐OP and f) LCPS‐CP after the addition of ALP. g) HPLC charts chromatograms of LCPS solutions with or without the addition of ALP. The LCPSs were incubated with 10 U mL^−1^ ALP for half an hour.

All the designed LCPS peptides, including phosphorylated and phosphonated peptides (Figure 1), were prepared by using Fmoc‐based solid‐phase peptide synthesis (SPPS) and characterized by analytical high‐performance liquid chromatography (HPLC) and electrospray ionization mass spectrometry (ESI‐MS) (Figure S1, Supporting Information). Fourier‐transform infrared (FTIR) spectroscopy results (**Figure** **2**a) indicated the characteristic peaks of some chemical groups (C═O in amide bonds (1670, 1570 cm^–1^) and C═O in carboxyl groups (1610–1550 cm^–1^)). Both LCPS‐OP and LCPS‐CP spectra showed the characteristic peaks of P═O in phosphate and phosphonate groups at 976.5 cm^–1^, while LCPS‐OH spectra did not have such peaks.

Subsequently, these three peptides were assembled in an aqueous solution under various conditions, and structural analysis was performed. The morphologies of the three LCPS peptide assemblies were further observed under transmission electron microscopy (TEM, Figure 2b). We found that three peptide assemblies that formed at the same pH value showed distinct morphologies. LCPS‐OH assemblies were rigid scattering fibrils with diameters of 200–500 nm at pH 3.0, 5.0, and 7.0 but interlaced networks at pH 9.0. In contrast, LCPS‐OP and LCPS‐CP displayed flexible fibrils with thinner diameters of 3–7 nm at pH 3.0 but interlaced network structures at pH 5.0, 7.0, and 9.0. Meanwhile, the LCPS peptide assemblies showed similar morphology in PBS and Dulbecco's modified eagle medium (DMEM) (Figure S3, Supporting Information).

Based on the measured zeta potential (Figure S4, Supporting Information) and the estimated isoelectric points (pI values, Table S1, Supporting Information) of the three peptides, we deduced that charge effects might induce different morphologies of the three peptide assemblies. The estimated pI values of the three LCPS peptides were 5.24 (LCPS‐OH), 2.41 (LCPS‐OP), and ≈3.54 (LCPS‐CP) because of different side chain modifications at the Ser site, as shown in Figure 1. At pH 7.0, the zeta potentials of LCPS‐OP and LCPS‐CP were −43.77 ± 1.89 and −26.37 ± 3.16 mV, respectively, more negative than that of LCPS‐OH (Figure 2c). The deprotonated phosphate or phosphonate group caused stronger intermolecular repulsion than the nonphsophorylated hydroxyl group due to charge effects, thus weakening the LCPS peptide assembly capability and inducing the production of an interlaced network structure instead of the large scattered fibrils that LCPS‐OH formed under the same conditions.

The secondary structures played important roles in the self‐assembly of the peptides. As shown in their CD spectra (Figure 2d, Figure S5, Supporting Information), at pH 3.0 and pH 7.0, all of the peptides had a similarly weak negative peak at ≈218 nm (*β*‐sheet structure). However, the sharp positive peak at 230–236 nm (exciton coupling between aromatic side chains)^[^ ^17^ ^]^ of LCPS‐OH was significantly stronger than those of LCPS‐OP and LCPS‐CP, indicating that the bulky phosphate and phosphonate groups weakened the interactions between Tyr side chains to result in the formation of flexible fibrils at pH 3.0 or an interlaced network at pH 7.0. In addition, a deconvolution of the amide I region in the FTIR spectra^[^ ^18^ ^]^ (Figure S6, Supporting Information) indicated that the three LCPS peptides had different ratios of secondary structures; the *β* sheet amount in the LCPS‐OH structure was higher than those in the LCPS‐OP and LCPS‐CP structures, demonstrating that phosphorylation and phosphonation changed the secondary structures of LCPS peptides and potentially affected the morphologies of their self‐assemblies.

The reversible self‐assembly properties of LCPS peptides are critical for their future application. Thus, we examined the effects of temperature on the assembly of the three peptides. Many LARKSs, including segment ^312^NFGTFS^317^ from the TDP‐43 protein^[^ ^19^ ^]^ and segments ^37^SYSGYS^42^ and ^54^SYSSYG^59^ from the FUS protein,^[^ ^13^ ^]^ formed reversible fibrils upon temperature variation. We found that the fibrils of LCPS‐OH formed at pH 7.0 completely disassociate as the temperature increased from 4 to 70 °C, upon cooling down to 4 °C, they form an interlaced network, instead of the initial scattered fibrils (Figure S7, Supporting Information). Both phosphorylation and phosphonation prevented complete dissociation of the fibrils of LCPS‐OP and LCPS‐CP with increasing temperature. Even at 70 °C, the fibrils transformed into only interlaced aggregates. As LCPS‐OP and LCPS‐CP were cooled to 4 °C, the fibrils regenerated. Thus, we deduced that phosphorylation and phosphonation at Ser^39^ affected the reversible assembly of the LCPSs but that LCPS‐OP and LCPS‐CP still maintained their reversible transformation between compact fibrils and interlaced network structures, indicating that their aggregates had relatively higher stability than LCPS‐OH aggregates. These results were consistent with the effect of phosphorylation on the mutant ^312^NFGAFS^317^ segment from TDP‐43, which initially exhibited reversible aggregation. After Ala^314^ was mutated to pThr^314^, the resultant ^312^NFGpTFS^317^ displayed irreversible aggregation.^[^ ^19^ ^]^

Notably, there is usually a high content of ALP in the physiological biomineralization environment.^[^ ^20^ ^]^ LCPS‐CP, in contrast to LCPS‐OP, was designed to resist hydrolysis by ALP while maintaining the regulatory effect of phosphate on peptide assembly and its mediated mineralization. Thus, it was necessary to evaluate the influence of ALP on the assembly and mineralization of phosphorylated/phosphonated LCPSs. First, we examined the morphologies of the two peptide assemblies in the presence of ALP. As shown in Figure 2e,f, hydrolysis of phosphate by ALP induced a morphological transformation of LCPS‐OP from interlaced networks to nanofibrils, which was similar to the structures of unmodified LCPS‐OH assemblies (Figure 2b, pH 7.0). In contrast, the phosphatase‐resistant LCPS‐CP assemblies maintained their interlaced network morphology after treatment with ALP. Next, we measured the stability of phosphate groups of two peptide assemblies (LCPS‐OP and LCPS‐CP) in the presence of ALP. Phosphate group of LCPS‐OP was completely hydrolyzed by ALP to release phosphate, while LCPS‐CP's remained intact (Figure 2g).

### 2.2 Evolution of the Initial Mineralization Products of the LCPS Peptides

The self‐assembly properties of the LCPS peptides encouraged us to further examine their mineralization. Bones and teeth utilize collagen fibrils as the primary matrix and use phosphoproteins (NCPs) to regulate the biomineralization process, which includes attraction of Ca^2+^ ions, clustering of the mineral ion, nucleation of amorphous CaP (ACP), crystal growth and transformation into major HAP organic‐inorganic nanocomposites.^[^ ^5^ ^]^ Thus, we further investigated the mineralization of these LCPSs in modified simulated body fluid (mSBF, 1.35 × 10^−3^ m CaCl_2_, 0.81 × 10^−3^ m K_2_HPO_4_, pH 7.0) as a medium. In general, collagen mineralization in natural hard tissues involves the formation of ACP as a precursor phase and subsequent transformation into HAP. Therefore, it was essential to investigate the effects of the LCPS‐OP and LCPS‐CP templates on the evolution of the mediated mineralization process. Herein, we used a real‐time UV–vis spectrometer to monitor the mineralization process and employed TEM and selected area electron diffraction (SAED) to detect the morphological transformation of mineralization products.

**Figure 3:**
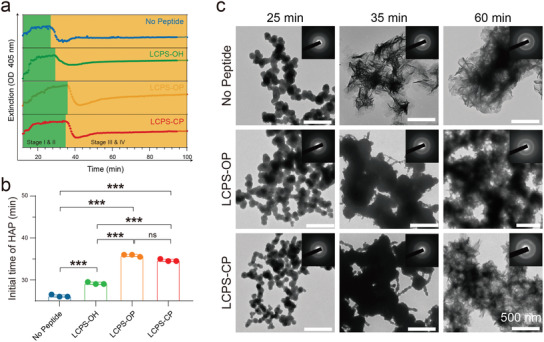
Evolution of mineralization mediated by phosphorylated/phosphonated LCPS peptides. a) Extinction curves (405 nm) of CaP solutions in the presence of different LCPS templates at stages I (appearance of ACP), II (aggregation of ACP), III (transformation of ACP to HAP), and IV (further growth and aggregation of HAP crystallites). Stages I and II and Stages III and IV are divided by different background colors (the boundary between green and orange indicates the initial HAP time). b) Statistics of the initial HAP time from (a). The data are the means ± SD (*n* = 3 repeats per group). The *P*‐values were determined by one‐way ANOVA, ***P < 0.001. c) Phase and morphology evolution of CaP minerals. TEM images and SAED patterns for the No Peptide, LCPS‐OP, and LCPS‐CP groups at 25, 35, and 60 min. Scale bar: 500 nm.

Based on the real‐time UV–vis extinction curves at 405 nm for mineralization mediated by different LCPS assemblies (**Figure** **3**a), all of the mineralization processes had variation tendencies similar to those in previous reports.^[^ ^21^ ^]^ The variations included four stages: i) appearance of ACP; ii) aggregation of ACP, during which the extinction curve of the solution increased and gradually reached a plateau; iii) transformation of ACP to HAP, during which the extinction curve suddenly decreased; and iv) further growth and aggregation of HAP crystallites.^[^ ^21^ ^]^ Nevertheless, phosphorylation and phosphonation of LCPSs delayed the beginning of stage III, which occurred at 35.83 ± 0.29 min for the LCPS‐OP group and 34.67 ± 0.29 min for the LCPS‐CP group in contrast to 26.17 ± 0.29 min for the No Peptide group and 29.17 ± 0.29 min for the LCPS‐OH group (Figure 3b). Thus, we concluded that all of the LCPS peptide templates extended the evolution of mineralization products from ACP to HAP and that phosphorylation and phosphonation enhanced the extension. The X‐ray diffraction (XRD) spectra in vitro biomimetic mineralization products of LCPS‐OP and LCPS‐CP after 30‐min incubation indicated typical broad peaks of stable ACP (Figure S8, Supporting Information). In contrast, the control group (No Peptide) at the same time point of mineralization produced typical HAP peaks (002, 211). These results were in accordance with previously reported results indicating that phosphorylated and phosphonated polymer hydrogels containing ALP and a phosphoric acid source can stabilize the ACP minerals formed in a controllable manner and enhance the mechanical strength of mineralization products.^[^ ^2b^ ^]^

Next, we measured the morphologies of three mineralized samples (No Peptide, LCPS‐OH, LCPS‐OP, and LCPS‐CP samples) by using TEM at different time points (Figure 3c and Figure S9, Supporting Information). We observed dynamic morphological changes in the different samples that were consistent with the UV–vis extinction results. At 25 min, the three mineralization samples displayed similar ACP morphologies. Gradually, the No Peptide mineralization sample transformed from ACP to a typical HAP morphology, as observed at 35 min, while the LCPS‐OP and LCPS‐CP mineralization samples underwent slower processes, displaying tail structures emerging from the formed ACP deposits at the same time point. In addition, the prolonged and crosslinked tails were different from the structure of the No Peptide CaP mineralization sample, which first formed a polygonal structure, as previously reported.^[^ ^21^ ^]^ Finally, the two LCPS‐mediated mineralization samples transformed to a typical HAP structure at 60 min. Thus, we concluded that the phosphorylated/phosphonated LCPSs regulated the initial evolution of CaP mineralization.

### 2.3 Long‐Term Mineralization Products of the LCPS Peptides

**Figure 4:**
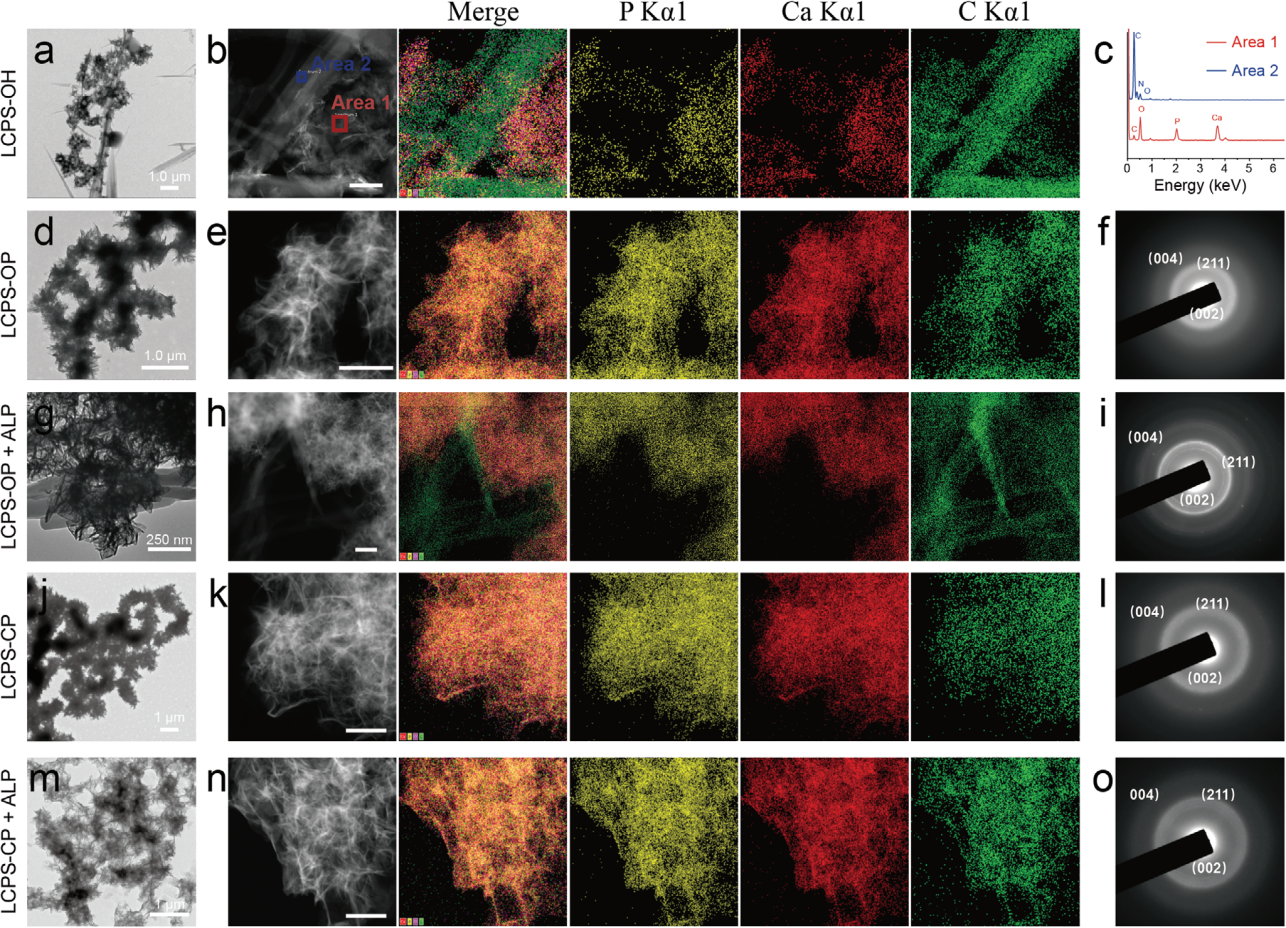
Mineralization of the phosphorylated/phosphonated LCPS peptide assemblies. Unstained TEM images showing partially mineralized a) LCPS‐OH and g) LCPS‐OP + ALP and completely mineralized d) LCPS‐OP, j) LCPS‐CP, and m) LCPS‐CP + ALP. Element mapping of mineralized b) LCPS‐OH, e) LCPS‐OP, h) LCPS‐OP + ALP, k) LCPS‐CP, and n) LCPS‐CP + ALP. c) EDS showing the element distribution of Areas 1 and 2 from (b). The SAED patterns of panels (f, i, l, o) match those of HAP and indicate oriented crystallization of mineral crystals.

The superior regulation of initial mineralization processes by the LCPS peptides prompted us to exhaustively investigate the long‐term mineralization products after 24 h of incubation. As shown in the TEM image of mineralized LCPS‐OH (**Figure** **4**a), a few CaP aggregates were attached to the scattering peptide nanofibrils, perhaps because the carboxyl group at the C‐terminus of LCPS‐OH serves as a nucleation site to initialize the formation of CaP minerals by attracting Ca^2+^ ions. However, element mapping results showed that the carbon element dots representing the LCPS‐OH organic scaffold had a completely different distribution area from the calcium and phosphorus element dots representing the CaP mineral phase, indicating attachment of CaP onto peptide fibrils rather than complete integration of CaP into the fibrils (Figure 4b). Energy dispersive X‐ray spectroscopy (EDS) analysis was performed, as shown in Figure 4c. Areas 1 and 2 of Figure 4c showed that completely different element mappings were obtained. In addition, we found that both LCPS‐OH and No Peptide mineralization led to similar loose CaP cluster microstructures (Figure 3a and Figure S10, Supporting Information).

In contrast, we observed substantial superimposition of the CaP mineral phase with the interlaced networks of peptides in the element mapping for the LCPS‐OP and LCPS‐CP mineralization products (Figure 4e,k). These results demonstrated that the LCPSs with phosphate or phosphonate modification were much better templates for biomimetic mineralization than native LCPS‐OH. The TEM morphological analysis described above indicated that LCPS‐OP and LCPS‐CP could form loosely interlaced network structures in contrast to the compact and scattering amyloid‐like fibril structure of LCPS‐OH, and these loose structures might facilitate subsequent biomineralization. HAP is the precursor phase for bone and tooth formation. The SAED patterns showed that the mineral phases were HAP‐like (Ca_10_(PO_4_)_6_(OH)_2_) with typical 002 and 112 crystallographic planes (Figure 4f,l).

Next, the influence of ALP on the mineralization of LCPS‐OP and LCPS‐CP was examined. The mineralization of LCPS‐OP was similar to that of LCPS‐OH in the presence of ALP (Figure 4g–i), which hydrolyzed the phosphate group of LCPS‐OP. However, mineralization of LCPS‐CP upon the addition of ALP involved fewer changes than mineralization of LCPS‐OP (Figure 4m–o) due to the ALP resistance of the phosphonate group, demonstrating the mineralization advantages of LCPS‐CP over LCPS‐OP.

### 2.4 Mediation of Tooth Enamel Remineralization by the LCPS Peptides

**Figure 5:**
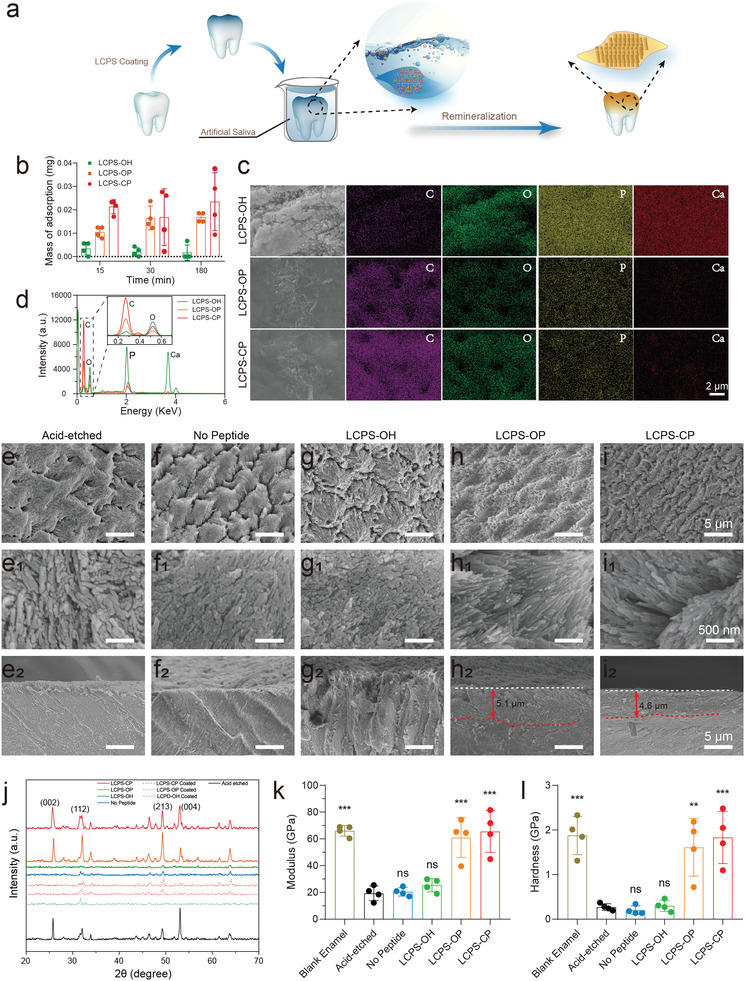
Remineralization and repair of demineralized enamel by LCPS coating. a) Scheme of the enamel remineralization procedure including LCPS coating of enamel and remineralization of enamel in artificial saliva. b) Mass of LCPS coated on enamel at different time points (15, 30, and 180 min). The enamel slices were immersed in 25 × 10^−6^ m LCPS solution. The data are the means ± SD (more than *n* = 3 repeats per group). c) Morphology and element mapping of enamel slices coated with LCPS; scale bar: 2 µm. d) EDX spectra of enamel slices coated with LCPS. The enamel slices were incubated with LCPS solution for 12 h in both (c) and (d). SEM low‐magnification images, high‐magnification images, and cross‐sectional images of acid‐etched enamel (e, e_1_, e_2_), No Peptide‐coated enamel (f, f_1_, f_2_), LCPS‐OH‐coated enamel (g, g_1_, g_2_), LCPS‐OP‐coated enamel (h, h_1_, h_2_), and LCPS‐CP‐coated enamel (i, i_1_, i_2_). Scale bars: 5 µm (e–i), 500 nm (e_1_–i_1_), and 5 µm (e_2_–i_2_). j) XRD spectra of HAP crystals on different enamel surfaces, including blank enamel, acid‐etched enamel, enamel coated with different LCPSs, No Peptide‐treated demineralized enamel, and LCPS‐treated demineralized enamel after 6 d of incubation in artificial saliva. k) Elastic modulus and l) surface hardness of blank enamel, acid‐etched enamel, No Peptide‐treated demineralized enamel, and LCPS‐treated demineralized enamel after 6 d of incubation in artificial saliva. The data are the means ± SD (*n* = 4 tooth slices per group, five measurements per sample, every data point was the mean of five measurements). The *P*‐values were determined by one‐way ANOVA, **P < 0.01, ***P < 0.001.

Because of the excellent performance of LCPS‐OP and LCPS‐CP in mediating in vitro mineralization, we examined the potential application of these LCPSs in tooth enamel remineralization and repair (**Figure** **5**a). Bovine defective enamel slices were fabricated, etched with phosphoric acid and then immersed in solutions of LCPS assemblies. We observed that the adsorption amounts of LCPS‐OP (16.76 µg) and LCPS‐CP (23.45 µg) were over tenfold greater than that of LCPS‐OH (1.67 µg) after 30 min of incubation (Figure 5b). In addition, the surfaces of enamel slices incubated with LCPS‐OH remained were covered mainly by inorganic HAP rods; only small areas had an organic layer, as shown in the SEM images (Figure 5c). In contrast, the enamel slices coated with LCPS‐OP or LCPS‐CP showed a dense organic film, which was also determined by SEM element mapping. The LCPS‐OP and LCPS‐CP‐coated enamel surfaces had many more carbon element dots and fewer calcium/phosphorus element dots than the LCPS‐OH‐coated enamel surface, consistent with the EDS results (Figure 5d). Thus, phosphorylation and phosphonation on LCPSs enhanced the deposition of LCPSs on the inorganic HAP surfaces of enamel slices.

After phosphoric acid etching, the enamel showed a porous and disordered structure because of the demineralization and deconstruction of the compact enamel layers (Figure 5e,e_1_,e_2_). In addition, XRD spectra exhibited strong diffraction peaks of HAP at 25.8° (002), 32.0° (112), 49.3° (213), and 53.1° (004), probably due to the presence of residual HAP (Figure 5j). Then, demineralized enamel slices coated or not coated with LCPS assemblies were exposed to remineralization buffer (2.58 × 10^−3^ m CaCl_2_·2H_2_O, 1.55 × 10^−3^ m, KH_2_PO_4_, 1 mg L^−1^ NaF, 180 × 10^−3^ m NaCl, 50 × 10^−3^ m Tris‐HCl, pH 7.6). After 24 h of incubation, inductively coupled plasma optical emission spectrometry (ICP‐OES) calculations showed significant gains in Ca and P for all the LCPS groups compared with the No Peptide‐treatment group (Figure S11, Supporting Information), indicating that the LCPS peptides endowed enamel with the ability to capture Ca^2+^ and PO_4_^3–^. After 3 d of incubation, all of the demineralized enamel groups (not coated with peptide or coated with LCPS‐OH, LCPS‐OP, or LCPS‐CP) formed undesirable and disordered structures in remineralization buffer (Figure S12, Supporting Information). In addition, although new HAP crystals were produced on the surface of enamel coated with LCPS‐OP or LCPS‐CP, as proven by XRD spectra (Figure S13, Supporting Information), their disordered and relatively disorganized morphologies were not as good as those of natural enamel. We considered that this might have resulted from the uncontrolled growth of HAP crystals due to spontaneous CaP precipitation in the remineralization buffer.

Accordingly, the remineralization buffer was replaced with commercial artificial saliva to mimic the real oral environment. After 3 days of incubation, the high‐magnification images of LCPS‐OP/CP groups revealed the initial formation of a new mineral layer with order microstructure compared with the etched enamel and other groups (Figure S14, Supporting Information). However, 3 d of incubation in artificial saliva could not lead to great remineralizaiton of enamel with compact, dense, and ordered HAP layers as the natural one. Furthermore, after 6 d of incubation in artificial saliva, the demineralized enamel slices coated with LCPS‐OP (Figure 5h, h_1_,h_2_) or LCPS‐CP (Figure 5i, i_1_,i_2_) displayed compact, dense, and ordered HAP layers, yielding XRD peaks identical to those of the original acid‐etched enamel. The uniform distribution of rod‐like HAP crystals on the surface greatly mimicked the “fish scale‐shaped” texture of natural enamel. In addition, the cross‐sectional images also clearly revealed the repaired enamel layers mediated by LCPS‐OP/CP (Figure 5h _2_,i_2_) in contrast to the destroyed layer on the top surface of acid‐etched enamel (Figure 5e _2_). The XRD spectra showed that the diffraction peaks of LCPS‐OP‐ and LCPS‐CP‐remineralized enamel were almost identical to the original acid‐etched enamel window, but some of the relative intensities of the diffraction peaks were stronger, such as those of peaks 002, 112, and 213 (Figure 5j). In contrast, the enamel slices coated with LCPS‐OH (Figure 5g, g_1_,g_2_) or No Peptide (Figure 5f, f_1_,f_2_) hardly generated well‐organized HAP crystals, as reflected by the XRD spectra (Figure 5j). The FTIR spectra showed significant deposition of phosphate group on the surface of tooth enamel coated with LCPS‐OP/CP, which was in consistence with the blank enamel (Figure S15, Supporting Information). Thus, introduction of LCPS‐OP and LCPS‐CP on the enamel surface promoted remineralization, probably because these two peptides can gather Ca^2+^ and PO_4_^3–^ onto the enamel surface and delay the transformation of ACP to HAP, ultimately resulting in the formation of rod‐like structures of HAP crystals.

Mechanical properties are critical characteristics of repaired enamel, so these properties were measured by using nanoindentation assays.^[^ ^22^ ^]^ As shown in the diagrams, the load–displacement curves reflected the elastic modulus (*E*) and hardness (*H*) (Figure S16, Supporting Information). Natural enamel showed an *E* of 65.99 ± 3.979 GPa (mean ± SD) and an *H* of 1.875 ± 0.4273 GPa. After phosphoric acid etching, the etched enamel showed dramatic deterioration of mechanical properties, with an *E* of 19.33 ± 5.49 GPa and an *H* of 0.2710 ± 0.07443 GPa. Remineralization of the etched enamel in artificial saliva generated new HAP layers, which resulted in restoration and even enhancement of mechanical properties, as reflected by an *E* of 60.92 ± 14.91 GPa and an *H* of 1.608 ± 0.6448 GPa for the LCPS‐OP group and an *E* of 65.43 ± 15.57 GPa and an *H* of 1.831 ± 0.5852 GPa for the LCPS‐CP group. In contrast, the remineralized enamel in the No Peptide group (*E* = 20.45 ± 3.138 GPa, *H* = 0.1990 ± 0.09606 GPa) and the LCPS‐OH group (*E* = 25.37 ± 4.947 GPa, *H* = 0.2988 ± 0.1253 GPa) displayed weak mechanical strength (Figure 5k–l).

Next, the remineralization and repair of the whole tooth enamel mediated by the LCPS‐OP or LCPS‐CP peptide were investigated. For comparison, etched whole tooth enamel was divided into two parts: one part was coated with waterproof glue, and the other part was used for remineralization mediated by LCPS‐OP or LCPS‐CP (Figure S17b, Supporting Information). The remineralized part showed a “fish scale‐shaped” texture and dense rod‐like HAP crystals (Figure S17a,c, Supporting Information), while the etched enamel had a porous and disordered structure (Figure S17d, Supporting Information). The element mapping and EDS indicated that the Ca/P ratios of newly generated HAP on the enamel surfaces treated with LCPS‐OP (1.65) or LCPS‐CP (1.62) were very similar to that of natural HAP (1.67) (Figures S17e and S18, Supporting Information).

### 2.5 Antibacterial Adhesion of LCPS Peptides Coated on the Enamel Surface

**Figure 6:**
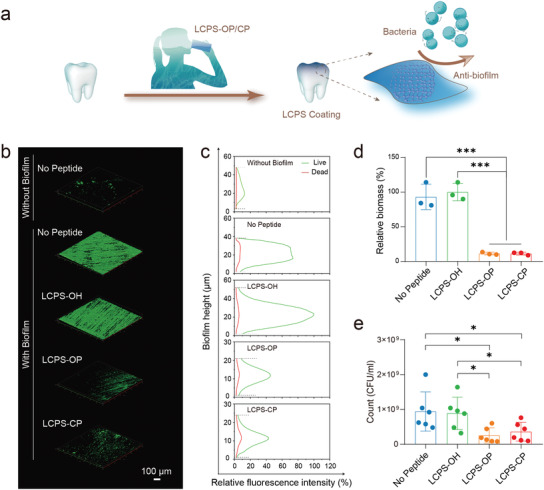
Antibacterial adhesion of enamel coated with LCPS in vitro. a) Schematic illustration of the coating of LCPS and its antibacterial adhesion properties. b) CLSM images of *S. mutans* biofilm distribution on the different enamels (without biofilm or with biofilm plus enamel coating with No Peptide, LCPS‐OH, LCPS‐OP, or LCPS‐CP). Scale bar: 100 µm. c) Distribution of the relative fluorescence intensities of live (green, labeled with SYTO 9) and dead (red, labeled with propidium iodide) *S. mutans* with the change in biofilm height. d) Relative biomass of enamel coated with No Peptide, LCPS‐OH, LCPS‐OP, or LCPS‐CP, derived from COMSTAT analysis of CLSM images of *S. mutans* biofilms. The data are the means ± SD (*n* = 3 repeats per group). The *P*‐values were determined by one‐way ANOVA, ***P < 0.001. e) CFU counts as a measure of the viability of *S. mutans* biofilms after 24 h of exposure to enamel coated with No Peptide, LCPS‐OH, LCPS‐OP, or LCPS‐CP. The data are the means ± SD (*n* = 6 repeats per group). The *P*‐values were determined by Student's *t*‐test, *P < 0.05.

Reducing the formation of bacterial biofilms on the tooth surface remains a significant challenge.^[^ ^23^ ^]^ Encouraged by the great capability of the LCPS peptides to mediate mineralization and enamel repair, we further investigated the antibacterial adhesion of the LCPS peptides to assess their potential application in hard tissue repair (**Figure** **6**a). First, enamel slices were incubated with each LCPS peptide. Then, they were incubated with planktonic *S. mutans*, the main cariogenic bacteria in the oral environment. The dispersal of the *S. mutans* biofilm on the enamel surface was evaluated with a live/dead assay and visualized by confocal laser scanning microscopy (CLSM) imaging. The LCPS‐OP and LCPS‐CP groups showed much lower intensities of green fluorescence (live bacteria) than the LCPS‐OH and No Peptide groups (Figure 6b,c), which was also reflected by the SEM images (Figure S19, Supporting Information). However, all of the groups showed very similar intensities of red fluorescence (dead bacteria), indicating that coating the enamel surface with LCPS‐OP or LCPS‐CP may inhibit the adhesion of bacteria but does not kill bacteria. In addition, the relative biomass values of the No Peptide and LCPS‐OH groups were more than eightfold greater than those of the LCPS‐OP and LCPS‐CP groups, as calculated by COMSTAT analysis (Figure 6d).^[^ ^24^ ^]^ The bacteria attached to the tooth enamel surface were dissociated by ultrasound and cultured for further counting. The groups with LCPS‐OP‐ and LCPS‐CP‐coated enamel had significantly lower colony‐forming unit (CFU) counts than the No Peptide and LCPS‐OH groups (Figure 6e and Figure S20, Supporting Information). Thus, we conclude that coating LCPS‐OP or LCPS‐CP onto the enamel surface can reduce bacterial adhesion, which is conducive to biomedical application of these peptides for tooth restoration.

### 2.6 Enhancement of Osteogenic Differentiation in the MC3T3‐E1 Cell Line

**Figure 7:**
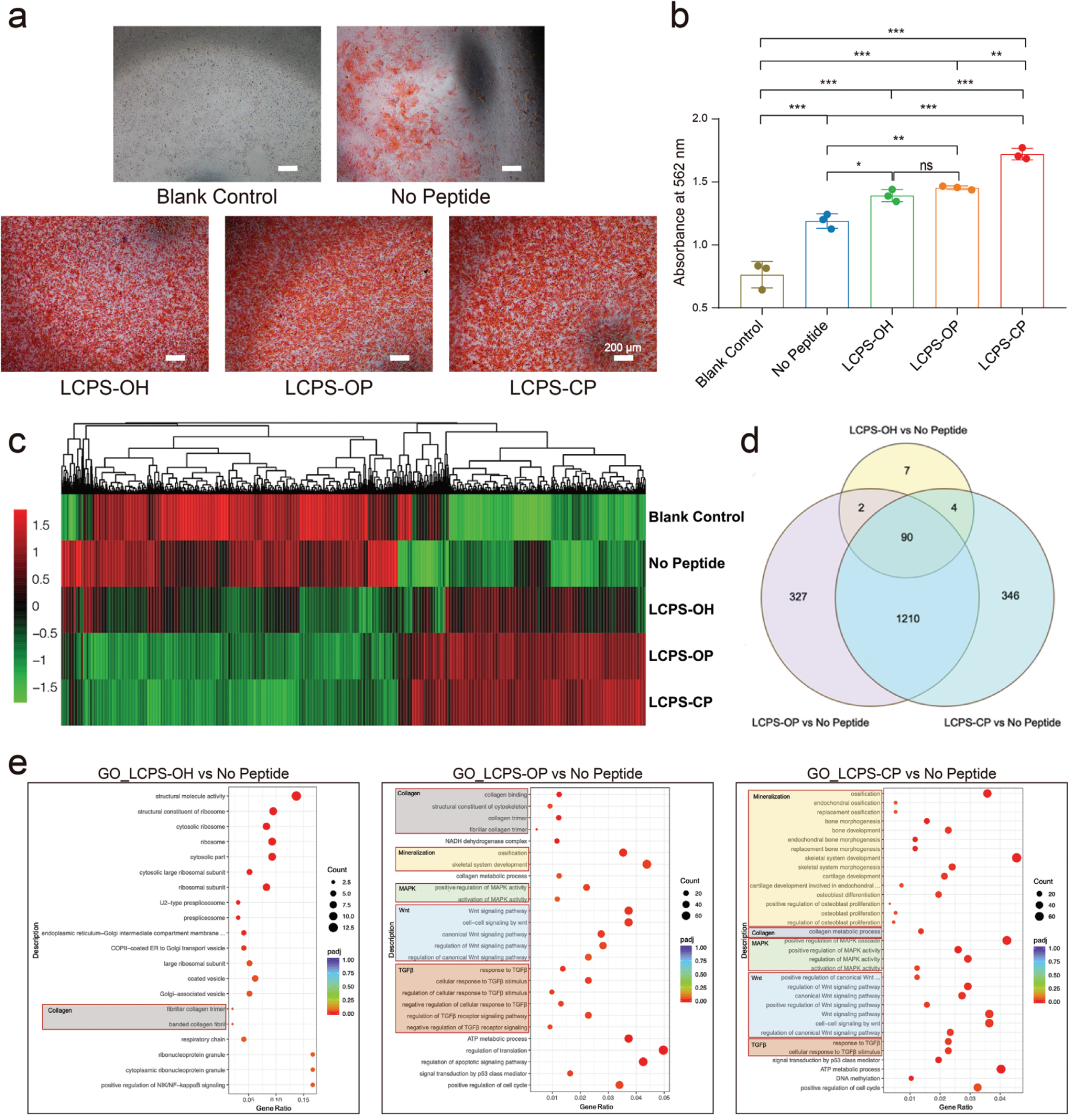
Osteogenic differentiation activity and transcriptome sequencing analysis of MC3T3‐E1 cells treated with LCPSs. a) Matrix mineralization of MC3T3‐E1 cells treated with LCPSs; scale bar: 200 µm. b) Quantitative analyses of extracellular matrix mineralization of MC3T3‐E1 cells cocultured with growth medium (blank control), differentiation medium (No Peptide), differentiation medium + LCPS‐OH (LCPS‐OH), differentiation medium + LCPS‐OP (LCPS‐OP), and differentiation medium + LCPS‐CP (LCPS‐CP). *n* = 3. The *P*‐values were determined by one‐way ANOVA, *P < 0.05, **P < 0.01, ***P < 0.001. c) Heat map of each group showing the fold changes (normalized value of FPKM) in the expression of selected genes. d) Venn diagram of differentially expressed genes among the No Peptide, LCPS‐OH, LCPS‐OP, and LCPS‐CP groups. e) Top regulated GO terms of MC3T3‐E1 cells after the addition of LCPS‐OH (left), LCPS‐OP (middle), and LCPS‐CP (right).

To further reveal the effects of the LCPS peptides on cell mineralization, MC3T3‐E1, a universal mouse preosteoblast cell line was selected as a model to explore their role in the osteogenesis. The cell viability results showed excellent cytocompatibility of the three LCPS peptide assemblies at concentrations of up to 50 × 10^−6^ m with MC3T3‐E1 cells from 24 to 96 h (Figure S21, Supporting Information). We also examined the osteogenic differentiation of MC3T3‐E1 cells cocultured with each LCPS peptide assembly by using alizarin red (AR) staining (**Figure** **7**a,b). The results of AR staining indicated that mineral deposition was greater in all of the LCPS groups than in the control group in the absence of peptides. In particular, the LCPS‐CP group exhibited the greatest mineral deposition and the brightest AR staining, which indicated that it exhibited the best extracellular mineralization after 16 days of incubation.

To further explore the effects of the LCPS peptides on the osteogenic signaling pathways, transcriptome sequencing (RNA‐seq) of MC3T3‐E1 cells incubated with each LCPS peptide assembly was carried out. After 2 d of incubation, characteristic gene expression profiles appeared in the LCPS groups, as revealed through cluster analysis. Slightly different characteristic gene expression was observed in the three treatment groups compared with the blank and No Peptide control groups (Figure 7c). Compared to the No Peptide control group, the LCPS‐OH, LCPS‐OP, and LCPS‐CP groups exhibited 103, 1629, and 1650 differentially expressed genes (DEGs, DESeq2 padj < 0.05), respectively (Figure 7d). Compared with the LCPS‐OH group, the phosphorylation group exhibited 1537 DEGs, and the phosphonation group exhibited 1556 DEGs (1883 DEGs were identified in total, including the unique and shared DEGs). The nearly 20‐fold difference indicated that there were significant differences in the responses of different genes to the stimulation of osteoblasts by modified and unmodified LCPSs with regard to mRNA levels. Moreover, gene ontology (GO) analysis and Kyoto Encyclopedia of Genes and Genomes (KEGG) pathway analysis demonstrated that mineralization‐related and signaling pathway‐related genes, such as genes involved in ossification, osteoblast proliferation, the Wnt pathway, the TGF‐*β* pathway, and the MAPK pathway were differentially expressed (Figure 7e and Figure S22, Supporting Information). Only pathways related to collagen growth or metabolism were differentially expressed in all LCPS groups compared with the No Peptide group (Figure 7e, left lane, gray box), suggesting an important effect on collagen metabolism. More biomineralization‐related pathways, such as the Wnt pathway (Figure 7e, middle and right lanes, blue box), the TGF‐*β* pathway (Figure 7e, orange box), and the MAPK pathway (Figure 7e, green box), were affected in the phosphorylated/phosphonated LCPS groups than in the LCPS‐OH group, with a few differences. Interestingly, the key mineralization‐related pathways (especially those related to the proliferation and differentiation of osteoblasts) were significantly enhanced in the LCPS‐CP group (Figure 7e, right lane, yellow box), indicating that there are slight differences in the mineralization‐promoting mechanisms of differently modified LCPSs. The mRNA levels of genes in these pathways were further confirmed by qPCR (Figure S23, Supporting Information). The results above show the biomineralization properties of LCPSs; specifically, they showed that phosphorylated/phosphonated LCPSs may influence osteogenic differentiation by regulating biomineralization‐related genes or signaling pathways.

## 3 Discussion

Biomineralization of bone and teeth is a complex process that requires synergistic effects of essential components in the microenvironment, including matrix proteins, regulatory additives such as hyperphosphorylated proteins, and osteogenesis‐related cells. Accordingly, the development of simplified biomimetic mineralized scaffolds combining the cooperative effects of matrixes, additives, and cell stimulation is necessary for the advancement of bone and tooth repair. Herein, we developed a new type of biocompatible peptide scaffold based on flexibly self‐assembling LCPSs containing a phosphate or phosphonate group. This is the first study to apply low‐complexity protein segments to achieve effective biomimetic mineralization. The two scaffolds (LCPS‐OP and LCPS‐CP) possess features distinct from those of previously reported self‐assembling mineralization templates (which often have rigid structures), including high aqueous solubility, structural flexibility, and the ability to form loose assemblies with interlaced morphology due to weak multivalent interactions. Therefore, they not only mimetically assemble matrix proteins but also likely function as regulatory additives to facilitate the formation of fused and merged hybrid composite of peptide and CaP mineral. Many proteins involved in the formation of bone and tooth, such as caseins, osteopontin, bone sialoprotein 2, and dentin dialophosphoprotein, contain indispensable disordered sequences for mediation of mineralization.^[^ ^25^ ^]^ Thus, LCPS‐OP and LCPS‐CP were further applied for tooth enamel repair, and they facilitated the epitaxial growth of HAP to generate new enamel comparable to the natural enamel and adapted to mitigate the adhesion of *S. mutans*, preventing the corrosive effects of *S. mutans* on enamel. Moreover, we revealed that LCPS‐OP and LCPS‐CP can stimulate osteogenic differentiation by activating some mineralization‐associated genes that regulate cells in mineralization microenvironments.

Regarding the mineralization mechanism of LCPS‐OP and LCPS‐CP, our results indicate that the phosphate and phosphonate groups anchored on the LCPS assemblies may be used as ion chelators to complex with Ca^2+^ and serve as mineralized templates for HAP. In addition, hydrophilic LCPS‐OP or LCPS‐CP can stabilize ACP and enhance wettability, which is critical for biomineralization. Natural collagen with a triple helix has a fiber structure and few acidic amino acids.^[^ ^26^ ^]^ Therefore, many extra molecules or ions, such as citrate,^[^ ^6^ ^]^ Mg^2+^,^[^ ^27^ ^]^ and hyperphosphorylated proteins^[^ ^5^ ^]^ are required to exert a wetting effect on biomineralization and regulate crystal growth. In contrast, hydrophilic LCPS‐OP and LCPS‐CP have relatively flexible structures and display dynamic self‐assembly properties, which likely generate excellent compatibility with CaP with the aid of the phosphate/phosphonate group. Thus, LCPS‐OP and LCPS‐CP play dual roles as both matrixes and regulatory additives to stabilize ACP during the mineralization process. In addition, we noticed that LCPS‐mediated mineralization in solution and on etched tooth enamel displays distinct temporal patterns. The mineralization process, particularly the formation of ACP and the transformation from ACP to HAP, requires much more time in the latter environment. We estimate that the mineralization reactions occurring on the interface between the solution and solid support (for remineralization of enamel) are much slower than those occurring in the solution phase.

Phosphonation, unlike phosphorylation, can resist hydrolysis by phosphatases enriched in biomineralization environments, which can help to maintain the assembly and mineralization properties of phosphonated LCPS in vivo. In addition, LCPS‐CP displays higher osteogenesis stimulation activity than LCPS‐OP, which might be due partly to phosphatase resistance and partly to the bioactivity of the phosphonate structure itself. Bisphosphonates (BPs), a class of commercial drugs used for the treatment of osteoporosis and similar diseases, have high binding affinity with bone minerals and exert stimulatory effects on the proliferation of osteogenesis‐related cells but inhibitory effects on osteoclasts.^[^ ^28^ ^]^ The indispensable bidentate phosphonate has been demonstrated to be the essential functional structure in all BP drugs.^[^ ^16^ ^]^ However, the side chains connected to the carbon atom can also affect the activity of BPs, which has led to the development of a series of BP drugs, such as alendronate and risedronate. Different side chains with distinctive charges can affect the total charge of BP, which potentially affects the pharmacological properties and binding with HAP.^[^ ^29^ ^]^ Due to its self‐assembly, the LCPS‐CP in this study had a high density of phosphonate groups on the surface, which might have been associated with its osteogenesis‐stimulating activity.

Numerous biocomposites with excellent mechanical properties are produced by biomineralization in tissues such as bone and teeth, of which the hardest is enamel.^[^ ^3a^ ^]^ However, dental caries is one of the most severe dental conditions. In 2010, more than 160 million children were affected by untreated caries, which was the tenth most prevalent health issue, particularly for children aged 1–4 years.^[^ ^30^ ^]^ The essential reason for dental caries is that bacterial fermentation of free sugars from food or juice can produce acidic by‐products that demineralize and destroy hard dental tissues.^[^ ^23^, ^30^ ^]^ As a result, both promoting remineralization and inhibiting demineralization are important strategies for the treatment of dental caries. On the one hand, LCPS‐OP and LCPS‐CP can be applied in tooth enamel regeneration and repair to produce new enamel layers with morphologies and mechanical properties comparable to those of natural enamel. In nature, dentin phosphophoryn (DPP), known as a “phosphate carrier,” is a critical protein of the dentin matrix. DDP functions as an inhibitor of crystal nucleation and growth in solution and as a template for crystal growth once immobilized on a solid surface, such as HAP or collagen fibrils.^[^ ^5^ ^]^ Therefore, we propose that the self‐assembling LCPS‐OP and LCPS‐CP can play DPP‐like roles in biomineralization systems. In addition, even though LCPS‐OP and LCPS‐CP are short peptides, their mineralization products display excellent mechanical properties, probably because they induce multiple levels of hierarchical organic–inorganic hybrid organization. On the other hand, demineralization can be inhibited by preventing the adhesion of oral bacteria associated with tooth decay. Therefore, the construction of antifouling enamel or teeth is a promising strategy to prevent dental caries. *S. mutans* is the main cariogenic bacterium in the oral cavity and easily adheres to the surfaces of teeth via hydrophobic interactions, calcium bridge static electricity and hydrogen bonding.^[^ ^31^ ^]^ In this study, the phosphate and phosphonate groups induced binding between LCPS‐OP or LCPS‐CP and the HAP of enamel, ultimately achieving excellent coating of LCPS on the tooth surface. Hydrophilic amino acids, such as Ser in LCPSs, endow LCPSs with considerable hydrophilicity, enabling the formation of an antifouling hydration shell and elimination of absorbed biomolecules.^[^ ^32^ ^]^ Moreover, the dense, negatively charged phosphate or phosphonate group coating on the enamel surface might cause electrostatic repulsion between the negatively charged bacterial membrane and the enamel, ultimately decreasing adhesion.^[^ ^33^ ^]^

Our further studies demonstrated that LCPS peptides can effectively stimulate osteogenic differentiation. Regarding the stimulation mechanism, the results of the transcriptome assay indicate that LCPS‐OP and LCPS‐CP can activate many signaling pathways in the MC3T3‐E1 cell line, including the Wnt, TGF‐*β*, and MAPK pathways. The Wnt signaling pathway is related mainly to embryonic development, tumorigenesis, and osteogenesis. Studies have shown that inhibiting Wnt signaling pathway transduction can hinder the differentiation of osteoblasts and inhibit bone formation, while the expression of Wnt family members can upregulate the expression of osteoblast‐specific genes and promote bone formation.^[^ ^34^ ^]^ The TGF‐*β* signaling pathway is another important signaling pathway that regulates the mineralization of osteoblasts and initiates the Smad signal transduction pathway to regulate the extracellular matrix synthesis of osteoblasts.^[^ ^35^ ^]^ Col1a1/Col3a1genes related to collagen fibril formation process rose to speak in cells treated by three types of LCPS peptide. Also, the expression levels of receptor genes Tgfbr3, Bmpr2, Bmpr1a and downstream transcriptional factor gene Sox9 were increased (Figure S23, Supporting Information), suggesting that LCPS peptide may upregulate the receptors and transcriptional factors expression in TGF*β*/BMP pathway, downstreamingly influence the target genes Col1a1/Col3a1 expression to affect the mineralization process. Interestingly, in contrast to the LCPS‐OP group, the LCPS‐CP group exhibited significant activation of the canonical MAPK signaling pathway. The MAPK signaling pathway plays important roles in tooth/bone development and metabolism. Of relevance to osteoblasts, the MAPK signaling pathway is the main contributor to osteogenic proliferation and differentiation.^[^ ^36^ ^]^ In the MAPK/ERK signaling pathway, RTKs such as the epidermal growth factor receptor activates Ras GTPase, which activates Raf. Raf activates MEK1/2, which further activates ERK1/2 acting on genes transcription. In our LCPS‐CP‐treated group, NF1 (the inhibitor of Ras‐GDP‐bound form) may downregulate, and RafB was up‐regulated to activate the MAPK/ERK pathway. This could explain why LCPS‐CP displayed the best performance in promoting mineralization.

## 4 Conclusion

In conclusion, these newly developed phosphorylated and phosphonated LCPS peptides display an excellent capability to mediate biomimetic mineralization, combining the synergistic effects of a template, regulatory additive, and osteogenesis stimulator. They have been successfully applied for remineralization and repair of etched tooth enamel, facilitating the generation of new enamel layers comparable to the natural layers and mitigating the adhesion of cariogenic bacteria to reduce corrosion risk. Our work not only provides a new biomineralization system based on LCPS but also sheds light on their potential biomedical applications in the regeneration and restoration of dental hard tissue.

## 5 Experimental Section

### Materials

Unless otherwise stated, the chemicals were purchased from Sigma‐Aldrich. All Fmoc‐amino acids, Fmoc‐Ser(tBuc)‐Wang Resin, 1‐hydroxy‐7‐azabenzotriazole (HOAt), and 2‐(7‐aza‐1H‐benzotriazole‐1‐yl)‐1,1,3,3‐tetramethyluronium hexafluorophosphate (HATU) were purchased from GL Biochem. Fetal bovine serum was purchased from Gibco. Minimum essential medium (MEM) Alpha Medium and Dulbecco's modified Eagle medium (DMEM) were purchased from Corning. Copper grid was purchased from Beijing Zhongjingkeyi Technology Co., Ltd. CellTiter‐Glo Luminescent Cell Viability Assay was purchased from Promega (Beijing) Biotech Co., Ltd. Alkaline phosphatase, calf intestinal (CIP, M0290) was purchased from NEW ENGLANK BioLabs Inc. Artificial saliva was purchased from Gladness Co. Ltd. BCA assay and LIVE/DEAD BacLight Bacterial Viability Kit (L7012) were purchased from Thermol Fisher Scientific Co. Ltd. Bovine tooth was commercially available and human tooth enamel samples were collected according to standard protocol for extraction at Peking University Hospital of Stomatology, handled with approval by the ethical committee of the hospital and agreed by the patients. The tooth samples window was cut using water‐cooled diamond saw. The work sides of samples were polished by silicon carbide paper. After that, all of samples were cleaned by ultrasonic treatment in deionized water for 20 min and stored in thymol solution (0.2 wt%) at 4 °C before use.

### Peptide Synthesis and Characterization

Synthesis of three peptides (LCPS‐OH, LCPS‐OP, and LCPS‐CP) was achieved by using standard Fmoc solid‐phase peptide synthesis manually. In short, Fmoc‐Ser(tBu)‐Wang resin (Loading: 0.295 mmol g^−1^, GL Biochem) was chosen for peptide anchoring. After the resins were swelling in DCM for 30 min, the Fmoc group was removed by treatment with 20% piperidine in DMF twice, respectively, for 5 and 15 min, producing free amino group. Next step was the coupling reaction between the amino group on the resins and the carboxyl group of Fmoc‐amino acid (4 eq.) purchased from GL Biochem for 1 h at room temperature in the presence of the coupling reagent 1‐hydroxy‐7‐azabenzotriazole (HOAt, 4 eq.), 2‐(7‐aza‐1H‐benzotriazole‐1‐yl)‐1,1,3,3‐tetramethyluronium hexafluorophosphate (HATU, 3.8 eq.), and the base N, N‐diisopropylethylamine (DIEA, 8 eq.). The above process was repeated to achieve the peptide elongation using commercial Fmoc‐protected amino acids except Fmoc‐protected phosphonated pSer mimetic that was prepared following previously reported methods.^[^ ^15a^ ^]^ After the completion of peptide coupling, the resins were dried in the vacuum for 2 h, followed by treatment with a solution of trifluoroacetic acid (TFA)/triisopropylsilane (TIS)/H_2_O (95/2.5/2.5, v) for 2 h to release the peptide, which was concentrated and then precipitated by cold diethyl ether. The crude peptide was then purified by reverse‐phase preparative HPLC (C18 column, SHIMADZU LC‐20A) and lyophilized. Finally, the obtained pure peptides were identified by analytical reverse‐phase analytic HPLC (C18 column, SHIMADZU LC‐2010A) and ESI‐MS (Thermo Fisher Ultimate 3000 Analytical and MSQ Plusinstrument).

### Preparation of LCPS‐OH Fibrils as well as LCPS‐OP and LCPS‐CP Interlaced Network

Peptides were dissolved in the ddH_2_O to a final concentration of 10 mg mL^−1^ by dropwise adding the 1 m NaOH solution. Then, the peptide solutions at different pH 1, 3, 5, 7, 9, and 11 were, respectively, prepared by adding hydrochloric acid solution. For the aggregation and assembly, the peptide solutions were incubated at 4 °C for 5 d.

### FTIR Spectroscopy

The FTIR spectra were measured by using a Frontier FTIR (PerkinElmer Life Sciences), which was equipped with an attenuated total reflectance assessor. All measurements were performed at room temperature. The peptide assembly solutions were directly dropped on the attenuated total reflectance assessor. After drying, the data were acquired with a resolution of 1 cm^–1^. The FTIR deconvolution of the amide I spectral region was conducted by Origin software.

### TEM for Morphology

The fibrils and interlaced network samples were directly deposited on a carbon‐coated copper grid for 45 s and then stained by using 1% uranyl acetate for 15 s. After drying, the TEM images were recorded by utilization of a Hitachi‐7650B electron microscope at 80 kV. The mineralization samples were directly deposited on a carbon‐coated copper grid for 40 min. After drying, the TEM images were recorded by utilization of a Hitachi‐7650B electron microscope at 80 kV directly.

### Zeta Potential Analysis

The zeta potentials of the aggregated peptides were analyzed by a Malvern ZEN3690 Zetasizer apparatus via traditional methods.

### Circular Dichroism Spectrum

The solutions of peptide assemblies were directly loaded in a 1‐mm light path CD cuvette. The CD spectra within the 190–260 nm region (far‐UV region) were measured with a Chirascan Plus CD spectrophotometer (Applied Photophysics). The signals were recorded at ambient temperature and three scans were averaged.

### Mineralization of LCPS Peptides

After assembly, 10 µL of the peptide assembly solution was diluted with equal volume of ddH_2_O. Then, 100 µL of the 1.35 × 10^−3^ m CaCl_2_ solution was added into the peptide assembly solution, and the resultant mixed solution was vibrated for 1 h to ensure the binding of calcium ions with the peptide aggregates. Next, 100 µL of the 0.81 × 10^−3^ m K_2_HPO_4_ solution was added dropwise for about 1 h while vibrating. The resultant solution was incubated at room temperature for the nucleation and growth of calcium phosphate. Mineralized samples of different time point was captured and detected by the TEM.

### UV–Vis Extinction Curves

The mineralization samples as mentioned above were incubated in the 96‐well plates, and the processes were monitored by using Synergy 4 plate reader. The absorbance at 405 nm was recorded to determine the evolution of the UV–vis extinction curves.

### HRTEM for the SAED Patterns and Energy‐Dispersive Spectroscope Mapping

The SAED patterns and energy‐dispersive spectroscope mapping (EDSM) were recorded using a high‐resolution transmission electron microscopy (HRTEM). The mineralization samples were deposited in the micro grid copper mesh for about 40 min. High‐resolution TEM images for the selected area electron diffraction were recorded by using JEOL JEM‐2100F. The elemental mapping and line scan were recorded on Oxford M‐max 80.

### Mineralization of LCPS Peptides in the Presence of ALP

The aggregated peptides were mineralized in the neutral condition directly as mentioned above, in the presence of 10 U mL^−1^ ALP (calf‐intestinal). After assembling, 10 µL of the peptide assembly solution was diluted with equal volume of ddH_2_O. Then, 100 µL of the 1.35 × 10^−3^ m CaCl_2_ solution containing 10 U mL^−1^ ALP was added into the peptide assembly solution, and the resultant mixed solution was vibrated for 1 h to ensure the binding of calcium ions with the peptide aggregates. Next, 100 µL of the 0.81 × 10^−3^ m K_2_HPO_4_ solution containing 10 U mL^−1^ ALP was added dropwise for about 1 h while vibrating. The resultant solution was incubated at room temperature for the nucleation and growth of calcium phosphate.

### Preparation of Demineralized Teeth

Tooth enamel slices and the whole tooth samples were etched with H_3_PO_4_ (37%) for 1 min and 10 min, respectively, to demineralize enamel and simulate early caries lesions. Then, all of the samples were transferred into deionized water, ultrasonicated for 20 min and dried in air.

### Preparation of Enamel Slices Coated with LCPSs

Self‐assembly LCPS (pH 7.0) were prepared as mentioned before. A solution of 25 × 10^−6^ m LCPS was diluted with physiological saline solution, respectively. Tooth enamel samples were plated on 24‐well plates and soaked into 2 mL of 25 × 10^−6^ m LCPS solution per well (*n* = 5). Concentration of the LCPS solution was monitored by BCA assay at 15, 30, and 180 min, respectively. After 12‐h incubation, washed with ultrapure water, and dried in air, the morphology of enamel slices was observed by SEM and the chemical elements mapping was measured by EDS. The adsorption amounts of LCPS were calculated by the initial concentration of LCPS minus detected concentration by BCA assay.

### LCPSs as Matrixes for Repair of Bovine Enamel Slices

Enamel slices were rinsed with deionized water and dried. The blank group and acid‐etched enamel were immersed in the artificial saliva or remineralization buffer. For the LCPS group, acid etched enamels were also immersed into artificial saliva or remineralization buffer after initial incubation in the LCPSs solution. After that, the remineralization occurred on the enamel slices in the artificial saliva or in the remineralization at 37 °C for 3 or 6 d. During the remineralization, the artificial saliva or remineralizaiton buffer in each sample was replaced once a day. In the end, the enamel slices were taken out, washed with ultrapure water three times, and dried in air for further characterizations.

### LCPSs as Matrixes for Human Whole Teeth Enamel Repair

For clear comparison, half of an acid‐etched tooth surface was immersed in the LCPS solution as the repair area for 12 h, and the remaining surface was exposed with air as the control. After coating of LCPS, the whole tooth was wash with ultrapure water and dried in air. After that, all of whole teeth were immersed in artificial saliva as same as the remineralization of enamel slices. After remineralization, the whole tooth was washed with ultrapure water and dried in air for further characterizations.

### Scanning Electron Microscopy

SEM images of remineralizaiton products were acquired by using a Hitachi SU8010 scanning electron microscope (Japan) with Pt sputtering. Both top‐down and side views of the sectioned tooth samples were observed in the same condition. EDS were collected using SU8010 equipped with an energy dispersive X‐ray spectrometer (Model 550i, IXRF Systems).

### X‐Ray Diffraction

X‐ray diffraction of each as‐prepared sample was measured by using XRD (SmartLab, Rigaku, Japan, with Cu K*α* radiation, wavelength = 0.154 nm). For power samples, the diffraction intensity was scanned in the 2*θ* range from 10° to 80° at acceleration voltage of 40 kV and a current of 150 mA. And for enamel slice samples, the diffraction intensity was scanned with a sampling step of 4° in the 2*θ* range from 20° to 70°, at an acceleration voltage of 45 kV and a current of 200 mA.

### Nanoindentation

The mechanical property of enamel samples was measured by a nanoindenter (G200, Keysight Technologie, CA, USA) with a Berkovich diamond tip (tip radius of ≈20 nm). Continuous stiffness measurement technique was applied for collecting the load–displacement curve of each enamel sample including blank enamel and acid‐etched enamel, no peptide coated demineralized enamel, and LCPS‐OH/OP/CP coated demineralized enamel at 25 °C with a relative humidity of 40%. For the test, the tip was calibrated with fused silica before evaluation. The constant strain rates were at 0.05 nm s^–1^ during the loading process. The depth with the limit of 1000 nm and load force were continuously monitored by the computer. The hardness and elastic modulus were obtained by calculating the mean value from 500 to 900 nm, and these data were presented as force–displacement curves.

### Culture of Bacterial Strains

*S. mutans* (UA159) were propagated in sterile brain heart infusion (BHI, CM1135) medium (3.7 g BHI power dissolved in 100 mL milliQ) in a sterile incubator containing 5% CO_2_ at 37 °C.

### Evaluation of Antibacterial Adhesion

All experimental operations are in sterile environment and materials were sterile. Enamels (5 mm × 3 mm × 1 mm) were, respectively, immersed in three different LCPS solutions (25 × 10^−3^ m) at 37 °C for 12 h (three enamel slices per group). After that, the enamels were taken out and washed three times with ddH_2_O. All of enamels were incubated at 12‐well plates. 1 mL of *S. mutans* in BHI (10^6^ CUF mL^−1^) was added into per well, using No Peptide coated enamels as controls. After 24‐h incubation at 37 °C, 5% CO_2_ incubator, the enamel slices were washed three times with ddH_2_O and transferred into a new 12‐well plate. The antibacterial adhesion activity was evaluated by CLSM, SEM, and viability counts. The SEM image was collected by the similar assay of remineralized enamel slices. Half of enamel samples per group were taken out and stained by mixed dye solution (1 mL ddH_2_O, 1.5 µL A solution (SYTO 9 dye, 3.34 × 10^−3^ m) and 1.5 µL B solution (propidium iodide, 20 × 10^−3^ m)) per well in dark for 15 min based on LIVE/DEAD BacLight bacterial viability assay. The other enamel slices were used for colony forming unit (CFU) counting. 1 mL bare BHI solution was added into each of the rest enamel samples. Separation of adherent *S. mutans* biofilm from the enamel surface was carried out by sonication for 5 min. The suspensions were diluted 10 000 folds and then seeded on horse blood agar. CFU was counted by automatic plater (easySpiral Pro Milk, Ref 413019) after 48‐h incubation.

### Confocal Laser Scanning Microscopy

Stained enamel samples were placed into the CLSM culture dishes (NEST, Cat. 801001) and observed by inverted CLSM (Zeiss LSM780). Specimens were illuminated by 488 nm (live colony) and 543 nm (dead colony) laser. Z‐stack images were obtained and captured by ZEN software. As described above, relative biofilm thickness was computed with COMSTAT 2 analyses in Image Software (www.comstat.dk).

### Cell Culture

MC3T3‐E1 cells were propagated in 89% alpha MEM with 10% FBS and 1% penicillin‐streptomycin solution. MG63 cells were propagated in 89% DEME with 10% FBS and 1% penicillin‐streptomycin solution. Both of these two cell lines were cultured in a sterile incubator containing 5% CO_2_ at 37 °C.

### Cell Viability Assay

Both MC3T3‐E1 cell line and MG63 cell line were plated on 96‐well plates at a density of 5000 cells/well (200 µL/well) in alpha‐MEM and DMEM with 10% fetal bovine serum cell culture medium and maintained at 37 °C and 5% CO_2_ atmosphere. After 24 h incubation at 37 °C, the medium was exchanged with fresh medium (100 µL, 100% alpha MEM and DMEM without fetal bovine serum) with LCPS assemblies of different concentrations (0, 10, 25, and 50 × 10^−6^ m). After 24, 48, and 96 h incubation following the culture protocol, a volume of CellTiter‐Glo Luminescent Cell Viability Assay equal to the volume of cell culture medium was added into each well. For inducing cell lysis, the mixture was put on an orbital shaker for 2 min. Cell viability was detected by recording luminescence in a Synergy 4 plate reader. At least three repeats per group.

### AR Staining

MC3T3‐E1 cell line was cultured in MEM with 10% FBS (growth medium) in 37 °C cell incubator with 5% CO_2_. To induce the MC3T3 cells differentiation, 10 × 10^−3^ m *β*‐sodium glycerophosphate, 50 µg mL^−1^ l‐ascorbic acid and 10 × 10^−9^ m dexamethasone were added to growth medium using as the differentiation medium. After culturing with differentiation medium and 25 × 10^‐6^ M LCPS peptides for 16 d and evaporation of excessive medium, the MC3T3 cells were fixed in 4% paraformaldehyde for 10–15 min. The fixative was discarded and the remaining was washed three times with ddH_2_O. After complete removal of the water, Alizarin Red S staining solution was slowly added into the fixed cells for detecting ECM mineralization for 20–30 min in 37 °C. The dye was then discarded and the remaining was washed with ddH_2_O 3–5 times, followed by imaging under a microscope (Zeiss, Germany). Finally, the samples dissolved by 10% cetylpyridinium chloride (CPC) were measured using a microplate reader at a wavelength of 562 nm.

### Transcriptome Sequencing of LCPS‐Treated MC3T3‐E1 Cells

After cultured with 25 × 10^‐6^ M LCPS for 2 d, total RNA of treated MC3T3‐E1 was obtained by TRizol^TM^ Reagent (Invitrogen, USA). RNA integrity was assessed using the RNA Nano 6000 Assay Kit of the Bioanalyzer 2100 system (Agilent Technologies, CA, USA). First strand synthesis reaction buffer, M‐MuLV Reverse Transcriptase (RNase H‐), DNA Polymerase I, AMPure XP system (Beckman Coulter, Beverly, USA), and Agilent Bioanalyzer 2100 system were used to prepare and assess the library. The clustering of the index‐coded samples was performed on a cBot Cluster Generation System using TruSeq PE Cluster Kit v3‐cBot‐HS (Illumia) according to the manufacturer's instructions. After cluster generation, the library preparations were sequenced on an Illumina Novaseq platform and 150 bp paired‐end reads were generated.

Raw data (raw reads) of fastq format were firstly processed through in‐house perl scripts. In this step, clean data (clean reads) were obtained by removing reads containing adapter, reads1 containing ploy‐N and low‐quality reads from raw data. At the same time, Q20, Q30, and GC content the clean data were calculated (shown in the Supporting Information). All the downstream analyses were based on the clean data with high quality. Reference genome and gene model annotation files were downloaded from genome website directly. Index of the reference genome was built using Hisat2 v2.0.5 and paired‐end clean reads were aligned to the reference genome using Hisat2 v2.0.5. FeatureCounts v1.5.0‐p3 was used to count the reads numbers mapped to each gene. And then FPKM of each gene was calculated based on the length of the gene and reads count mapped to this gene. Differential expression analysis of two conditions/groups (two biological replicates per condition) was performed using the DESeq2 R package (1.20.0).

GO enrichment analysis of differentially expressed genes was implemented by the clusterProfiler R package, in which gene length bias was corrected. GO terms with corrected *P*‐value less than 0.05 were considered significantly enriched by differential expressed genes. KEGG is a database resource for understanding high‐level functions and utilities of the biological system, such as the cell, the organism, and the ecosystem, from molecular‐level information, especially large‐scale molecular data sets generated by genome sequencing and other high‐through put experimental technologies (http://www.genome.jp/kegg/). clusterProfiler R package was used to test the statistical enrichment of differential expression genes in KEGG pathways. All sequencing results were submitted to the NCBI, Accession: PRJNA730673.

### RT‐qPCR

First, total RNA from treated MC3T3‐E1 cells was isolated by TRizol^TM^ Reagent (Invitrogen, USA). RNA was immediately converted to cDNA via a PrimeScritRT^TM^ reagent Kit (Takara, Japan). The products were used for amplified with TB Green Premix Ex TaqTM II (TAKARA, Japan) under a two‐step cycling condition using an LightCycler480II (Roche, Switzerland). Primer sequences used in the experiment are presented in Table S4 (Supporting Information).

## Conflict of Interest

The authors declare no conflict of interest.

## Author Contributions

R.C. and Y.J.L. contributed equally to this work. R.C. conceived the idea of the project, conducted synthesis and mineralization experiments, and drafted the manuscript. Y.J.L. conducted cell mineralization experiments and drafted the manuscript. R.C. and Y.L.Z. performed in vitro mineralization experiments and data analysis. R.C. performed the remineralization of teeth enamel experiments and characterization. S.Y.Z. helped collect tooth, performed the *S. mutans* experiments and data analysis. R.C. and B.B.H. performed cell viability experiments and analyzed data. F.C. and Y.X.C. supervised the project, designed the assays, and wrote the paper together with R.C. and Y.J.L.

## Supporting information

Supporting Information

## Data Availability

The data that support the findings of this study are available from the corresponding author upon reasonable request.
