# Supplementary material for: Phosphorylated and Phosphonated Low‐Complexity Protein Segments for Biomimetic Mineralization and Repair of Tooth Enamel
Source: Adv Sci (Weinh). 2022 Jan 2;9(6):2103829. doi: 10.1002/advs.202103829 (PMC8867149; doi:10.1002/advs.202103829)
Supplement: Supplementary file 1 — Supporting Information [file ADVS-9-2103829-s001.pdf]

WILEY-VCH

ADVANCED SCIENCE
Open Access

Supporting Information

for *Adv. Sci.*, DOI: 10.1002/advs. 202103829

Phosphorylated and Phosphonated Low-Complexity Protein Segments for Biomimetic Mineralization and Repair of Tooth Enamel

Rong Chang,† Yang-Jia Liu,† Yun-Lai Zhang, Shi-Ying Zhang, Bei-Bei Han, Feng Chen, Yong-Xiang Chen**

This file includes:

Figsure S1 to S23
Tables S1 to S4

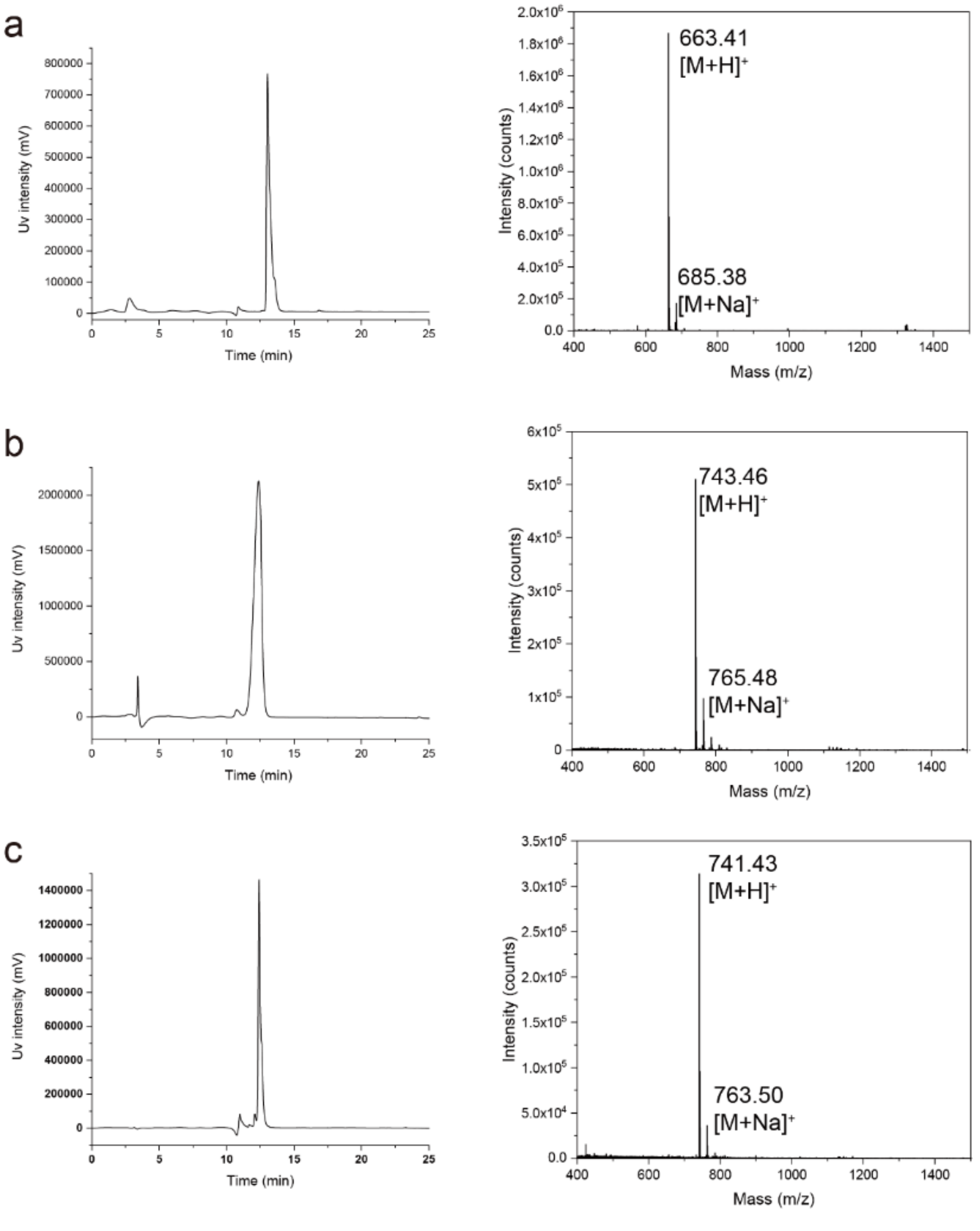

Figure S1. High-performance liquid chromatography (HPLC) and electrospray ionization mass spectrometry (ESI-MS) analysis of three LCPS peptides. (a) LCPS-OH, (b) LCPS-OP, (c) LCPS-CP.

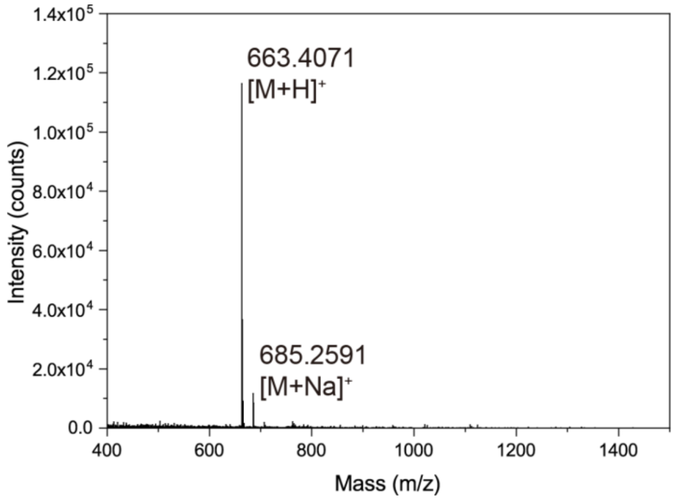

Figure S2. Electrospray ionization mass spectrometry (ESI-MS) analysis of LCPS-OP after treatment of ALP.

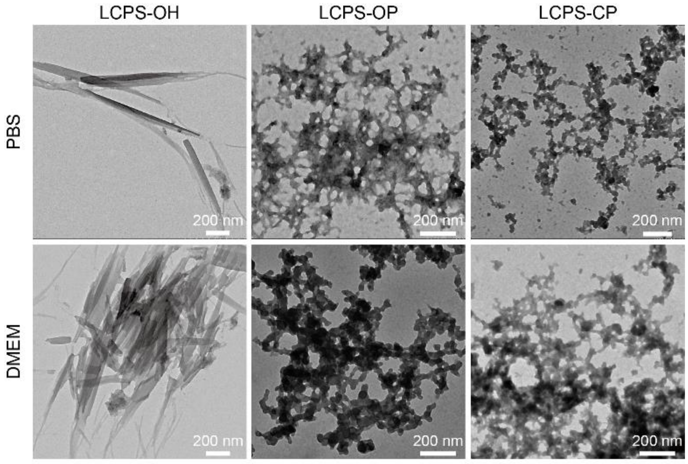

Figure S3. Negatively stained TEM images showing the self-assembled structures of LCPS-OH, LCPS-OP, and LCPS-CP in PBS and Dulbecco's modified eagle medium (DMEM). Scale bar: 200 nm.

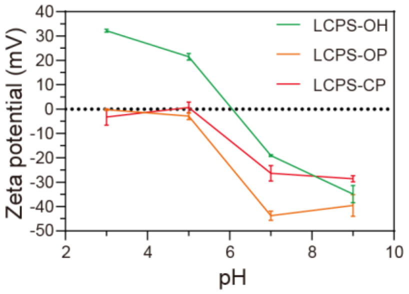

Figure S4. Zeta potential of LCPS-OH, LCPS-OP and LCPS-CP at different pH.

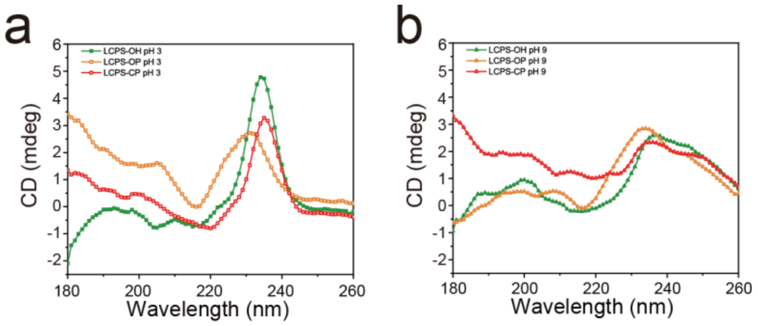

Figure S5. CD spectra of LCPS-OH, LCPS-OP, and LCPS-CP assembly solutions (10 mg/ml) at pH 3.0 (a) and pH 9.0 (b).

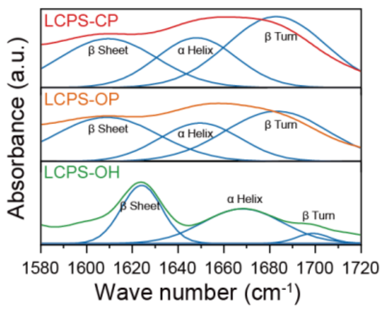

Figure S6. FTIR deconvolution of assembled LCPSs at amide I spectral region.

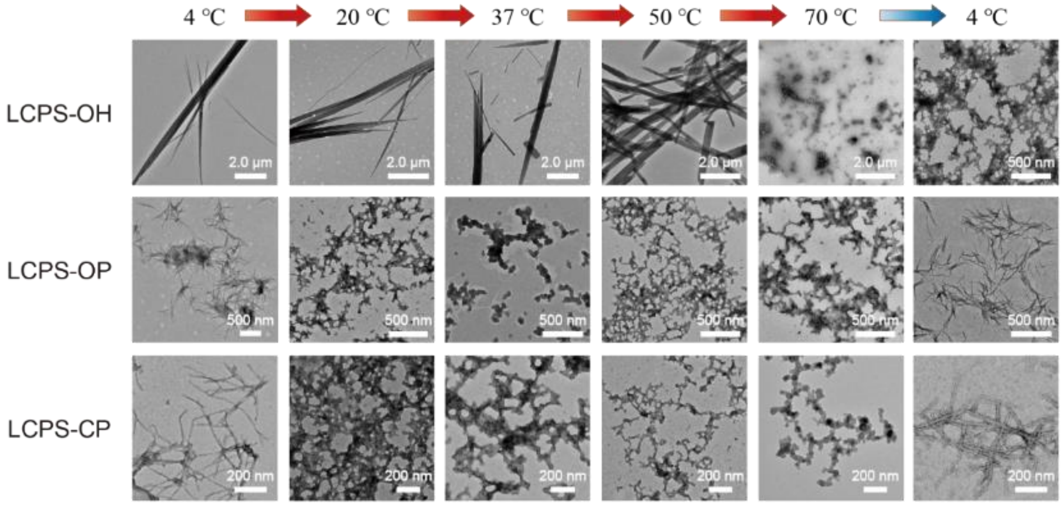

Figure S7. The effects of temperature on the assembly of three LCPS peptides characterized by TEM. Fibrils formed at 4 °C were warmed up to 20 °C for 1 h, then the temperature was increased to 37 °C for 1 h, 50 °C for 1 h, 70 °C for 1 h respectively. To reform the fibrils, samples were subsequently cooled down to 4 °C and incubated for 24 h. Each peptide was measured at least three times.

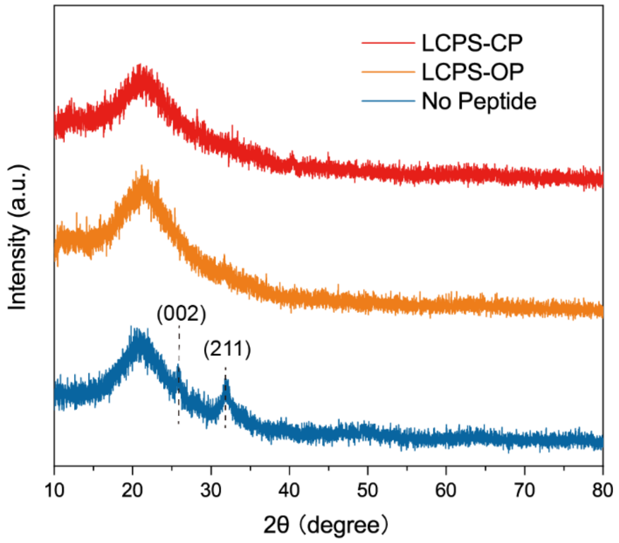

Figure S8. XRD spectra of the mineralized products of No Peptide, LCPS-OP and LCPS-CP for about 30 mins. The mineralization solution was centrifuged for 5 mins, washed with water, and then immediately frozen with liquid nitrogen and lyophilized for getting the solid sample to be analyzed.

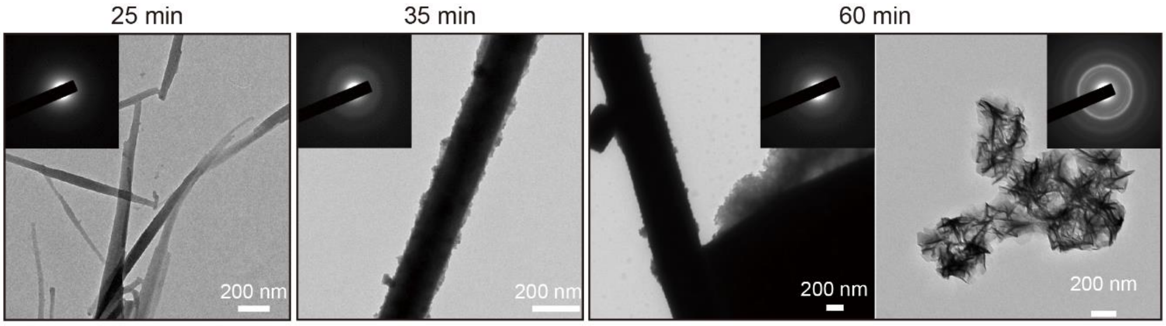

Figure S9. Phase and morphology evolution of CaP minerals. TEM images and SAED patterns for the LCPS-OH groups at 25 min, 35 min and 60 min. Scale bar: 200 nm.

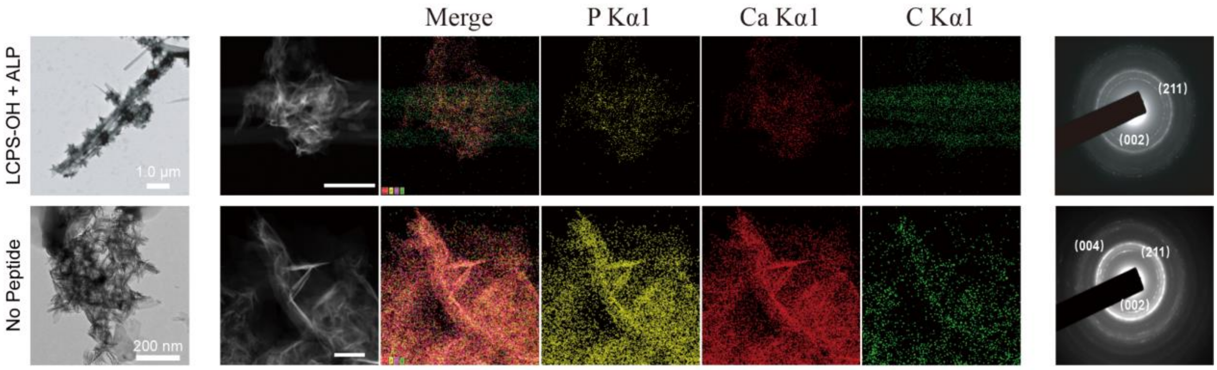

Figure S10. Unstained TEM images showing mineralized No Peptide and LCPS-OH + ALP. Element mapping of mineralized and the SAED patterns of panels.

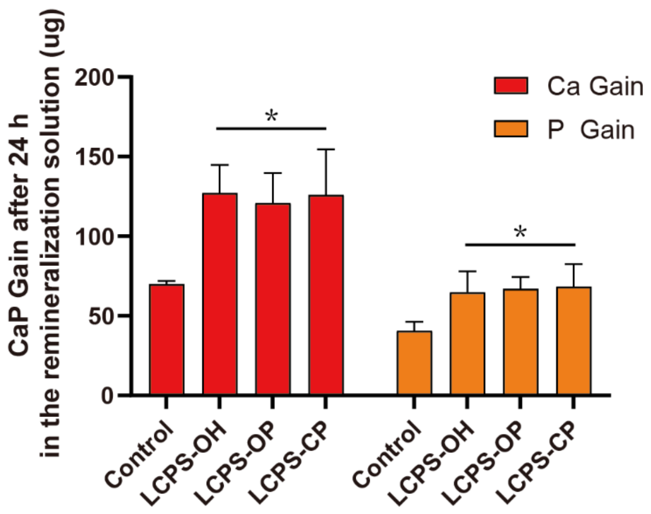

Figure S11. Mineral gain of enamel surfaces after 24-hours incubation in remineralizaiton solutions, analyzed by inductively coupled plasma optical emission spectrometry (ICP-OES). All LCPS groups showed significant difference compared with control group. The data are the means ± SD ($n = 3$ repeats per group). The P values were determined by Student's t test, $^{*}P < 0.05$.

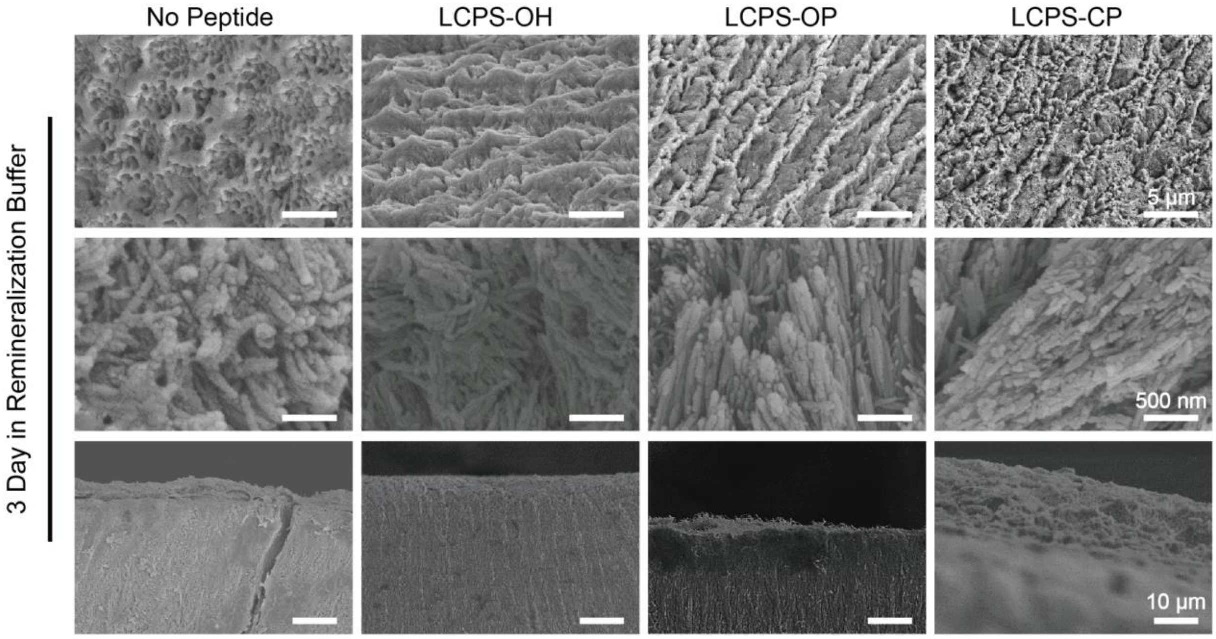

Figure S12. SEM low-magnification images, high-magnification images and cross-sectional images of No Peptide-coating enamel, LCPS-OH-coating enamel, LCPS-OP-coating enamel, and LCPS-CP-coating enamel after 3 days of incubation in remineralizaiton buffer.

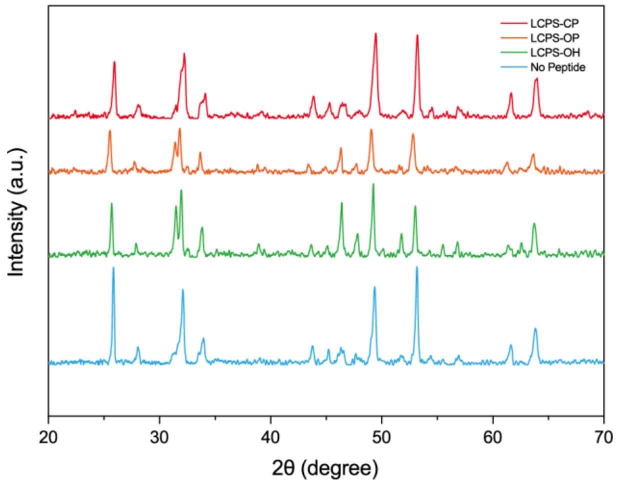

Figure S13. XRD spectra of HAP crystals on different enamel surfaces, including No Peptide-coating enamel, LCPS-OH-coating enamel, LCPS-OP-coating enamel, and LCPS-CP-coating enamel after 3 days of incubation in remineralizaiton buffer.

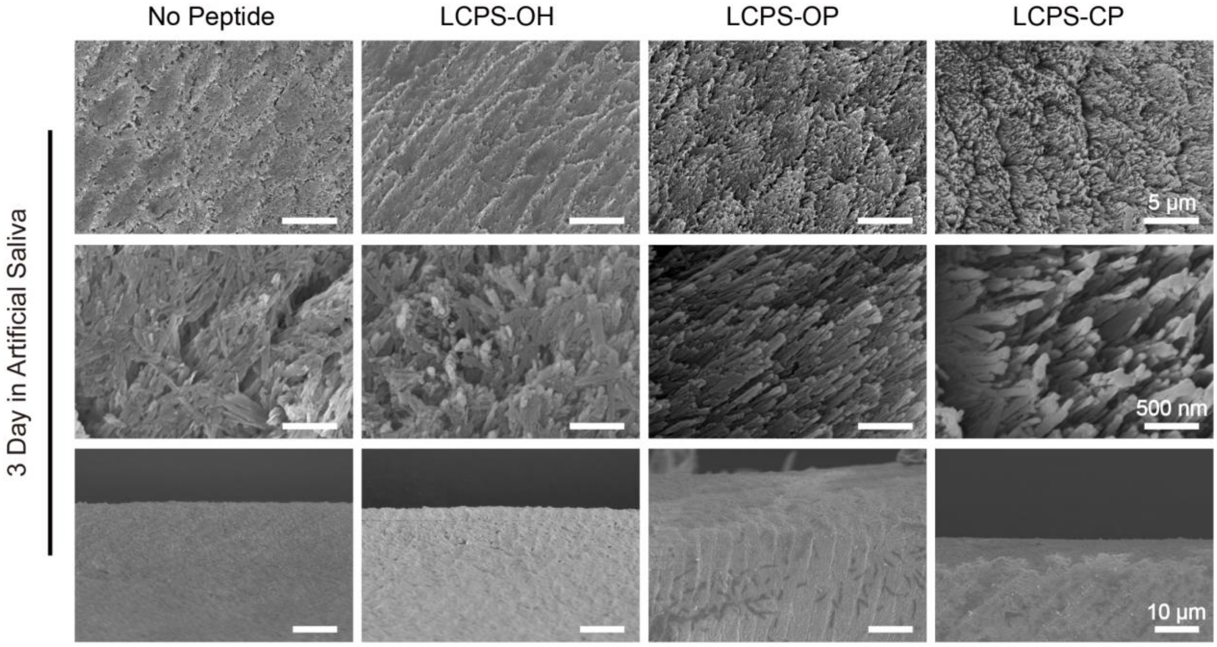

Figure S14. SEM low-magnification images, high-magnification images and cross-sectional images of No Peptide-coating enamel, LCPS-OH-coating enamel, LCPS-OP-coating enamel, and LCPS-CP-coating enamel after 3 days of incubation in artificial saliva.

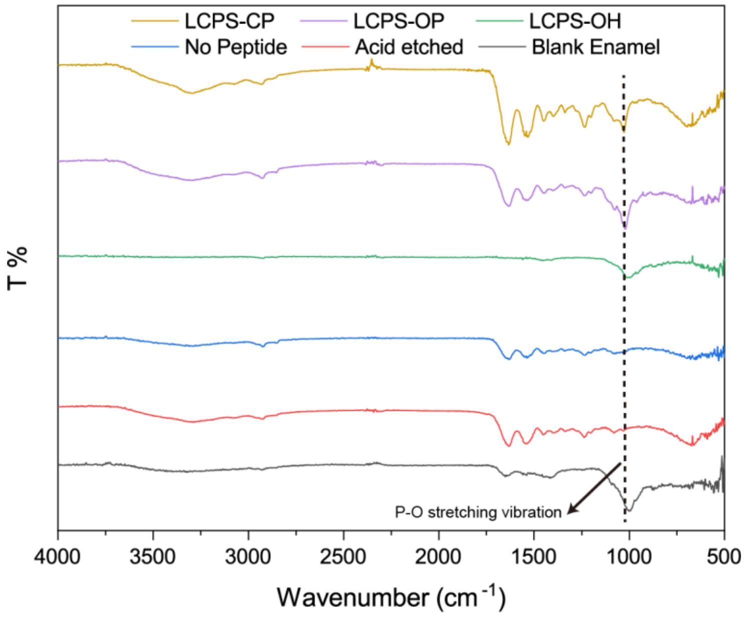

Figure S15. FTIR spectra of different enamel layers including blank enamel, acid-etched enamel, enamel coated with different LCPSs, No Peptide-treated demineralized enamel, and LCPS-treated demineralized enamel after 6 days of incubation in artificial saliva. P-O stretching vibration peaks around 1065 cm^{-1}.

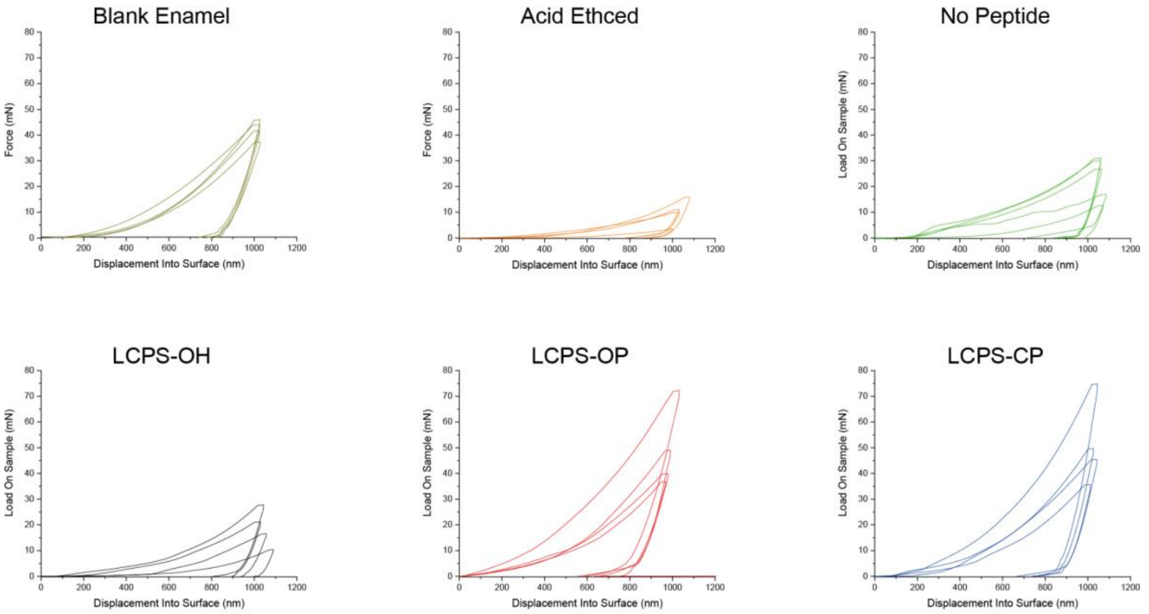

Figure S16. Loading–unloading curves of Nano-indentation tests including blank enamel, acid etched enamel and remineralized No Peptide-coating enamel, LCPS-OH-coating enamel, LCPS-OP-coating enamel, and LCPS-CP-coating enamel.

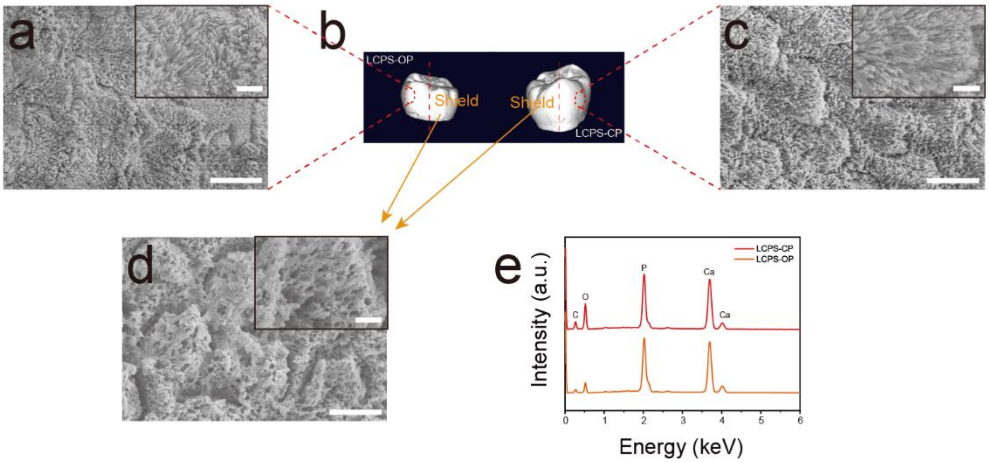

Figure S17. LCPS coating-induced remineralization of demineralized whole human teeth. a) SEM image of the demineralized whole human tooth surface with porous microstructure. b) SEM images of the remineralized and repaired surface morphology of demineralized whole human teeth coated with LCPS-OP. c) Microcomputed tomography (micro-CT) image of two acid-etched teeth with a half region remineralized by the LCPS-OP or LCPS-CP coating. d) SEM images of the remineralized and repaired surface morphology of demineralized whole human teeth coated with LCPS-CP. Scale bars, 5 μm (a-b, d) and 1 μm (a-b, inset, and c, inset). e) EDX spectra of HAP regenerated on remineralized whole human teeth coated with LCPS-OP (orange line) and LCPS-CP (red line).

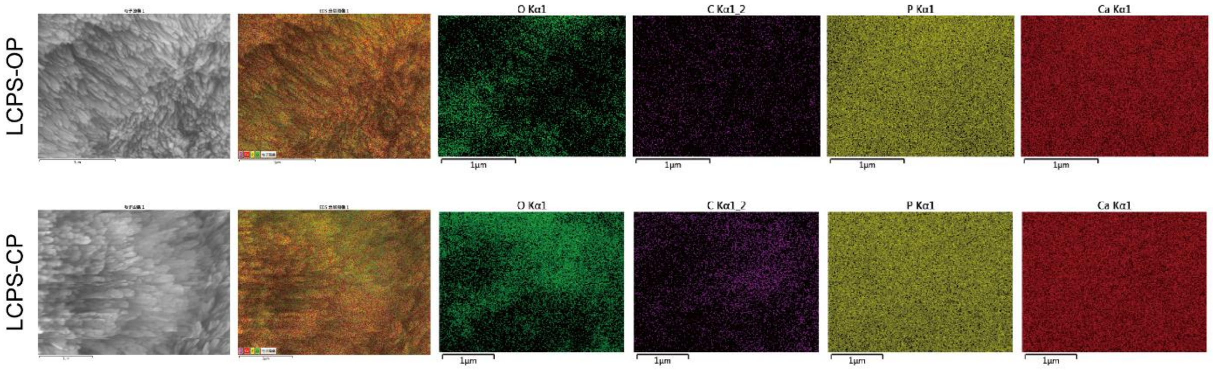

Figure S18. Element mapping of HAP regenerated on remineralized whole human teeth coated with LCPS-OP and LCPS-CP.

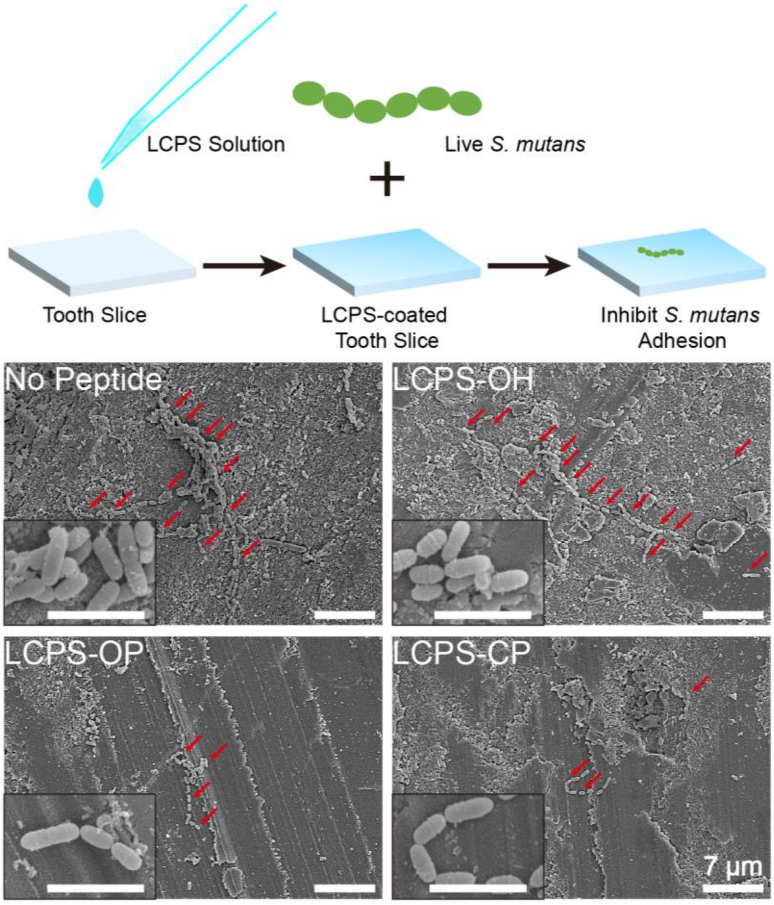

Figure S19. Anti-adhesion of *S. mutans* evaluated by SEM image. SEM images of S. mutans biofilm distribution after the incubation on the LCPS-coated tooth slices. Scale bars: 7 μm, 2 μm (inset). The red arrow refers to the *S. mutans*.

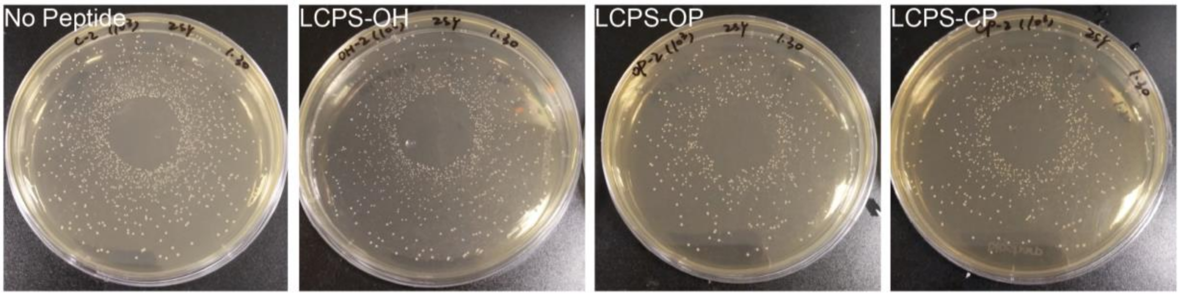

Figure S20. Representative culture plate images of *S. mutans* colonies dissociated by ultrasound and cultured for counting in different enamel groups including No Peptide-coating enamel, LCPS-OH-coating enamel, LCPS-OP-coating enamel, and LCPS-CP-coating enamel.

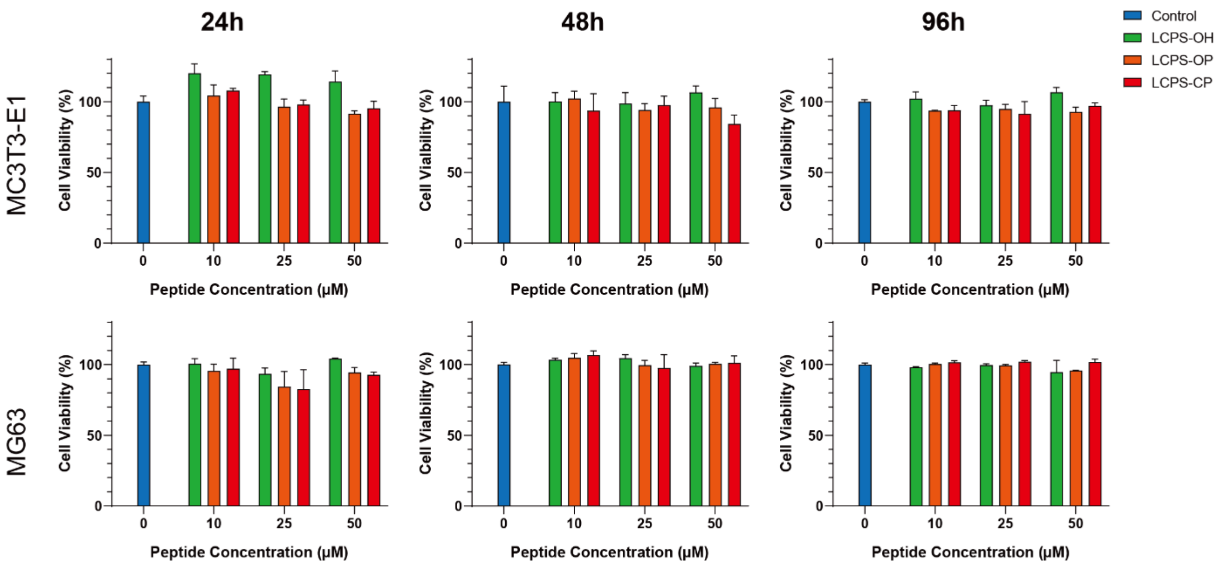

Figure S21. Cell viability of MC3T3-E1 and MG63 cell at different concentrations (0, 10, 25, 50 μM) of LCPSs for 24 h, 48 h and 96 h. The data are the means ± SD. (n=4)

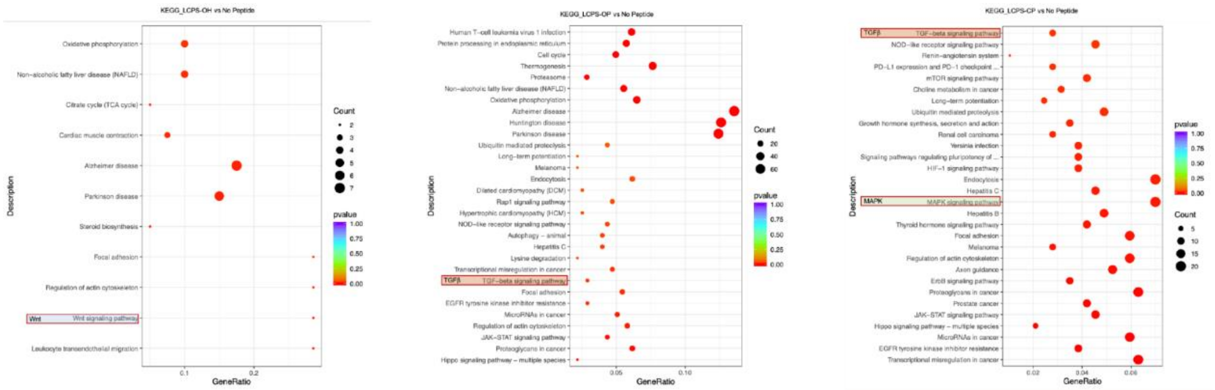

Figure S22. Top regulated KEGG terms of MC3T3-E1 after addition of LCPS-OH (left), LCPS-OP (middle), LCPS-CP (right).

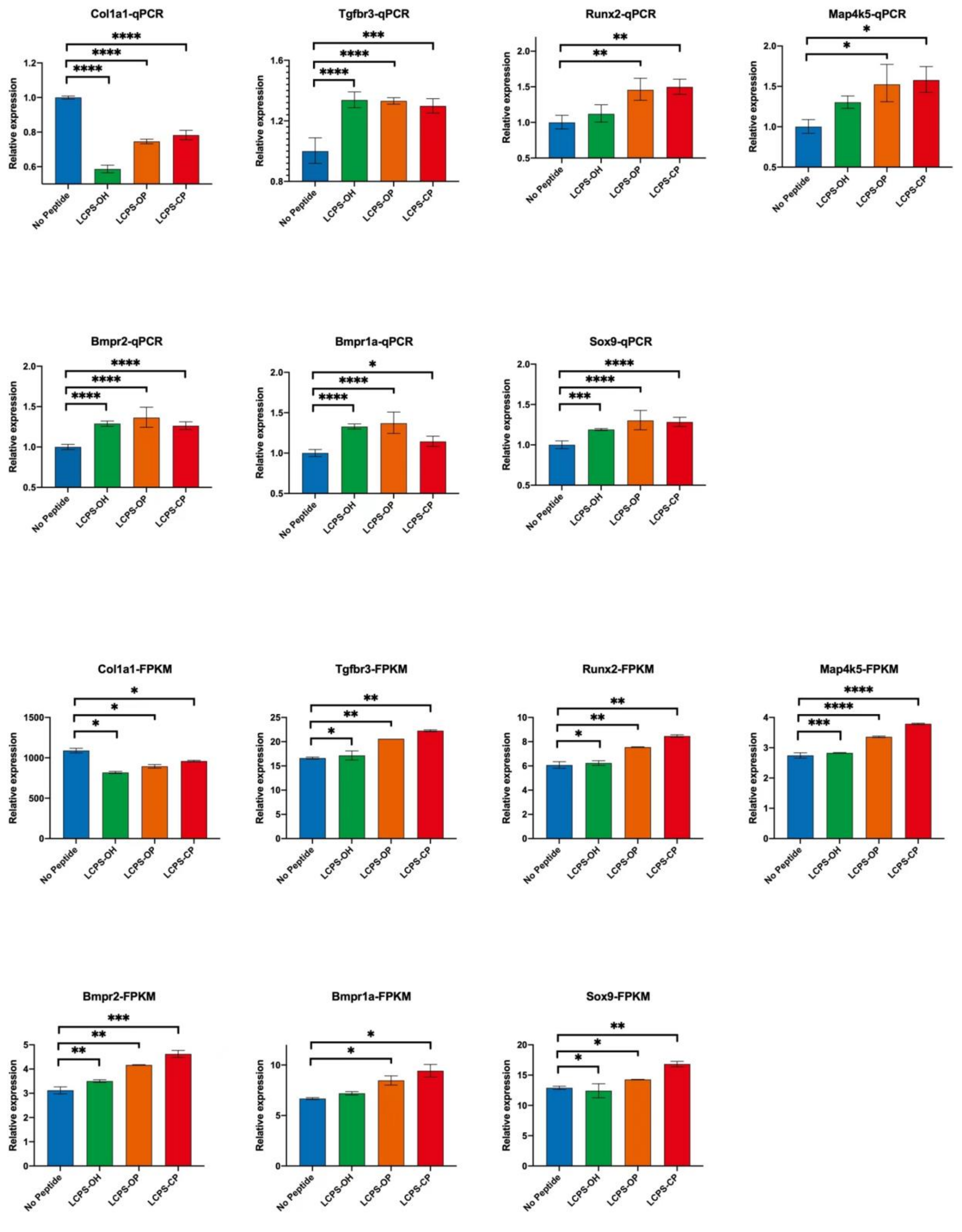

Figure S23. Gene expression of Runx2, Col1a1, Tgfbr3, Bmpr2, Bmpr1a, Sox9, Map4k5 in MC3T3-E1 treated with No peptide, LCPS-OH, LCPS-OP, LCPS-CP at day 2 by quantitative RT-PCR (upper) and FPKM(lower) from RNA-seq. The data are the means ± SD. n = 3. The P values were determined by One-way ANOVA, $*P < 0.05$, $**P < 0.01$, and $***P < 0.001$.

Table S1. Estimated isoelectric point (pI) and net charge at pH 7.0 of three peptides

Name	Estimated pI	Net charge at pH 7.0
LCPS-OH	5.235	-0.783
LCPS-OP	2.405	-2.543
LCPS-CP	~ 3.54	~ -1.98

Table S2. Summary of sequencing data quality. raw_reads: the number of reads in the raw data. clean_reads: the number of reads after filtering the original data. clean_bases: The number of bases after filtering the original data (clean base=clean reads*150bp). error_rate: the overall sequencing error rate of the data. Q20: The percentage of bases with a Phred value greater than 20 (Q20) or 30 (Q30) to the total bases. GC_pct: The percentage of G and C in the four bases in clean reads.

**Sample**	**Raw_reads**	**Clean_reads**	**Clean_bases**	**Mean length**	**Error_rate**	**Q20**	**Q30**	**Gc_pct**
**No peptide1**	46329438	44895324	6.73G	266.103898	0.02	98.43	95.21	51.42
**No peptide2**	45162774	43616164	6.54G	261.01545	0.02	98.33	95.01	50.75
**LCPS-OH1**	45406390	43855150	6.58G	258.418725	0.02	98.33	94.94	49.92
**LCPS-OH2**	46091168	44642588	6.7G	260.644698	0.02	98.37	95.04	50.12
**LCPS-OP1**	47030020	45489808	6.82G	258.229093	0.02	98.38	95.07	49.82
**LCPS-OP2**	46386444	44998284	6.75G	260.146706	0.02	98.4	95.12	49.82
**LCPS-CP1**	47534650	46273018	6.94G	260.322096	0.02	98.4	95.14	49.77
**LCPS-CP2**	45817008	44525560	6.68G	259.507559	0.02	98.42	95.19	49.68

Table S3. Differention gene numbers statistics.

Compare	all	up	down	threshold
LCPS-OHvsControl	103	22	81	DESeq2 padj<0.05 |log2FoldChange|>0.0
LCPS-OPvsControl	1629	678	951	DESeq2 padj<0.05 |log2FoldChange|>0.0
LCPS-CPvsControl	1650	698	952	DESeq2 padj<0.05 |log2FoldChange|>0.0

Table S4. Real time-PCR primer sequences.

Primers	Sequence
GAPDH-F	GGCCTTCCGTGTTCCTACC
GAPDH-R	TGCCTGCTTCACCACCTTC
RUNX2-F	CCAAGTAGCCAGGTTCAACG
RUNX2-R	GGTGAAACTCTTGCCTCGTC
Col1a1-F	GCTCCTCTTAGGGGCCACT
Col1a1-R	CCACGTCTCACCATTGGGG
Tgfbr3-F	GGTGTGAACTGTCACCGATCA
Tgfbr3-R	GTTTAGGATGTGAACCTCCCTTG
Bmpr2-F	TTGGGATAGGTGAGAGTCGAAT
Bmpr2-R	TGTTTCACAAGATTGATGTCCCC
Bmpr1a-F	AACAGCGATGAATGTCTTCGAG
Bmpr1a-R	GTCTGGAGGCTGGATTATGGG
Sox9-F	GAGCCGGATCTGAAGAGGGA
Sox9-R	GCTTGACGTGTGGCTTGTTC
Map4k5-F	CAACATCGTAGCCTACTTTGGG
Map4k5-R	GGTAGCTGTAATTTTTGCAGCCA
